# Six-fold reduction in ocean heat content estimate uncertainty since 1960

**DOI:** 10.1038/s41467-026-76436-0

**Published:** 2026-08-29

**Authors:** Huifeng Yuan, Lijing Cheng, Yuying Pan, Bin Zhang, Benoit Meyssignac, Kevin E. Trenberth, Yujing Zhu, Xinyi Song, Huayi Zheng, Senliang Bao, Juan Du, Jiang Zhu, Zhong Jin, Xuebin Chi, Jinrong Jiang, Rongwang Zhang, Yuan Tian, Ning Liu

**Affiliations:** 1 State Key Laboratory of Earth System Numerical Modeling and Application, Institute of Atmospheric Physics, Chinese Academy of Sciences, Beijing, China; 2 Computer Network Information Center, Chinese Academy of Sciences, Beijing, China; 3 University of Chinese Academy of Sciences, Beijing, China; 4 Institute of Oceanology, Chinese Academy of Sciences, Qingdao, China; 5 Oceanographic Data Center, Chinese Academy of Sciences, Qingdao, China; 6 Université de Toulouse, CNES, CNRS, IRD, UT3, LEGOS, Toulouse, France; 7 NSF National Center for Atmospheric Research, Boulder, CO USA; 8 Department of Physics, University of Auckland, Auckland, New Zealand; 9 College of Meteorology and Oceanography, National University of Defense Technology, Changsha, China; 10 International Center for Climate and Environment Sciences, Institute of Atmospheric Physics, Chinese Academy of Sciences, Beijing, China; 11 South China Sea Institute of Oceanology, Chinese Academy of Sciences, Guangzhou, China

**Keywords:** Ocean sciences, Climate change

## Abstract

Ocean heat content (OHC) dominates Earth’s energy imbalance, but its uncertainty has not been fully understood. Here, we construct a Large-Ensemble to comprehensively quantify the OHC uncertainty from global to regional scales since 1955, and consider eight groups of error sources based on the community’s efforts. The global upper 2000 m OHC estimated uncertainty (defined as 5–95% quartile error range) has reduced six-fold since 1955 from ~111 ZJ (1955–1965) to ~19 ZJ (2014–2023), mainly due to reduced mapping/sampling uncertainty. This indicates a robust upper 2000 m OHC increase since 1955 of 449 [385, 519] ZJ. Quality-Control (QC) has become a primary error source recently, due to the difficulties detecting outliers in eddy-rich regions. Trend uncertainty sources differ from annual OHC: for 2005–2023, the upper 2000m trend is 10.9 [9.2, 11.4] ZJ yr^−1^ (0.68 [0.57, 0.71] Wm^−2^), with uncertainties from spatial gaps, mapping, QC, and climatology choice. The ocean warming rate increase from 2005–2023 is small but robust: 0.34 [0.19, 0.46] Wm^−2^ dec^−1^. These comprehensive uncertainty estimates guide future improvements in the global ocean observing system.

## Abstract (web summary)

This study presents a comprehensive quantification of ocean heat content uncertainty since 1960 and shows a six-fold reduction in the global upper-2000m uncertainty in the last decade and a robust ocean warming.

## Introduction

Oceans store ~90% of Earth’s energy imbalance (EEI), making ocean heat content (OHC) change a critical climate indicator^1–4^. Observations show that the OHC has broken its warming record nine years in a row from 2017 to 2025 (ref. ^5^), and has a long-term acceleration since 1960^6–9^. The rapid ocean warming has become a significant concern for the ocean’s biological systems and for human society^3,10^. For example, thermal expansion contributes ~40% of the contemporary sea level rise and poses significant risks to low-lying coastal communities^2^. Ocean warming reduces the solubility of oxygen in the sea water and contributes to the ocean deoxygenation, threatening ocean life and marine fisheries^11^. Increase of ocean heat also super-charges tropical cyclones and increases their intensity and rainfall^12^. As an essential climate variable, OHC plays a crucial role in the interactions among Earth’s energy, water, and carbon cycles^3,13,14^.

Given its importance, substantial efforts have been made to estimate and understand OHC changes^3,4,9^, despite differences existing among different available OHC estimates^2–4,9,15^. For instance, a synthesis of 21 available datasets^15^ reveals that the trend of global OHC from 2005 to 2020 ranges from 0.40 to 0.96 Wm^−2^ with an acceleration rate from −0.03 Wm^−2^ dec^−1^ to 1.11 Wm^−2^ dec^−1^, revealing a substantial spread among the available estimates. Therefore, understanding the sources of errors and quantifying uncertainty is crucial to refining the OHC estimates over time and is important for improving the current global ocean observing system (GOOS). Additionally, as previous investigations reveal a critical mismatch of inter-annual variations between most in situ observation-based OHC estimates and net radiation imbalance (EEI) observed by satellite at the top of the atmosphere (TOA)^4,9^, understanding the sources of uncertainty will provide important clues for reconciling these EEI differences.

Several previous efforts have indeed been dedicated to comprehensively quantifying the OHC uncertainty, but have limitations of considering neither some important error sources nor the error covariances. The first comprehensive estimate of the OHC (0–700 m) uncertainty budget is Lyman et al.^16^, where four error sources from 1993 to 2008 were considered: combined quality-control and eXpendable BathyThermographs (XBT) bias correction, sampling, mapping, and climatology choice. That work identified XBT bias correction as the major source of error (with a maximum standard deviation of ~24 ZJ around 2000), whereas mapping played a secondary, but important, role (~9 ZJ).

As different approaches are used to estimate different error sources, inconsistencies occur and the uncertainties for each error source are not necessarily comparable. To resolve these issues, an international coordinated effort^16^ used a set of consistent sensitivity experiments to quantify errors from three sources separately: XBT bias correction, mapping, and the choice of climatology from 1970 to 2008. Several alternative methodologies were used for each, and then the standard deviation of their resultant OHCs was calculated as a quantification of uncertainty. The regional uncertainty was then investigated in a follow-on study with the same experiments^17^. The mapping method to fill in inevitable sampling gaps was identified as the primary source of error in OHC calculation (mean standard deviation of 16.5 ZJ from 1970-2008), and the XBT bias correction uncertainty ranged from 8.0 to 17.9 ZJ since 1970. However, the overall uncertainty has not been defined because only parts of error sources were considered and the error covariances were not analyzed.

Limitations of previous efforts have made it unclear what contributes most to the uncertainty of OHC estimates and what the total uncertainty is. OHC uncertainty comes mainly from the following ten error sources: instrumental error^18^, quality control procedure^19^, biases in various instruments (XBT^20^, mechanical bathythermographs -MBT^21^, Bottle—BOT^22^, Autonomous Pinniped Bathythermographs -APB^23^), vertical interpolation methods^24,25^, choices of climatology^26,27^, and data sampling/mapping^16,17,26,28–30^. Additionally, these uncertainty sources are not independent; for example, different QC systems flag and remove various amounts of data, which in turn impact ocean sampling and mapping accuracy. Furthermore, the uncertainty for different time scales (e.g., annual mean, inter-annual, decadal to multi-decadal changes, and long-term trends) should be different, as the errors are manifested differently at various time scales as the observing system has evolved or because different types of errors are correlated on different time scales (see results of this study). A comprehensive assessment of these error sources, accounting for error covariances, is the goal of this study.

To assess all the identified error sources and the error covariance, a Large-Ensemble approach is used for OHC uncertainty estimation. Briefly, for each major group of error sources (8 groups in total with 10 different error sources considered), we employed the three most up-to-date alternative techniques used in the literature for their estimation, and then these techniques were cross-used to derive final OHC estimates, allowing for a large ensemble to be constructed with ~900 members (Data and Methods). The ensemble is used to derive global and regional OHC uncertainty and the major contributors for different periods since 1955 (slightly before the International Geophysical Year when global OHC trend can be derived^3,31^). The uncertainty and the error sources for estimating the OHC annual mean, trend and variability are quantified separately.

## Results

### Global OHC and associated uncertainty

**Fig. 1:**
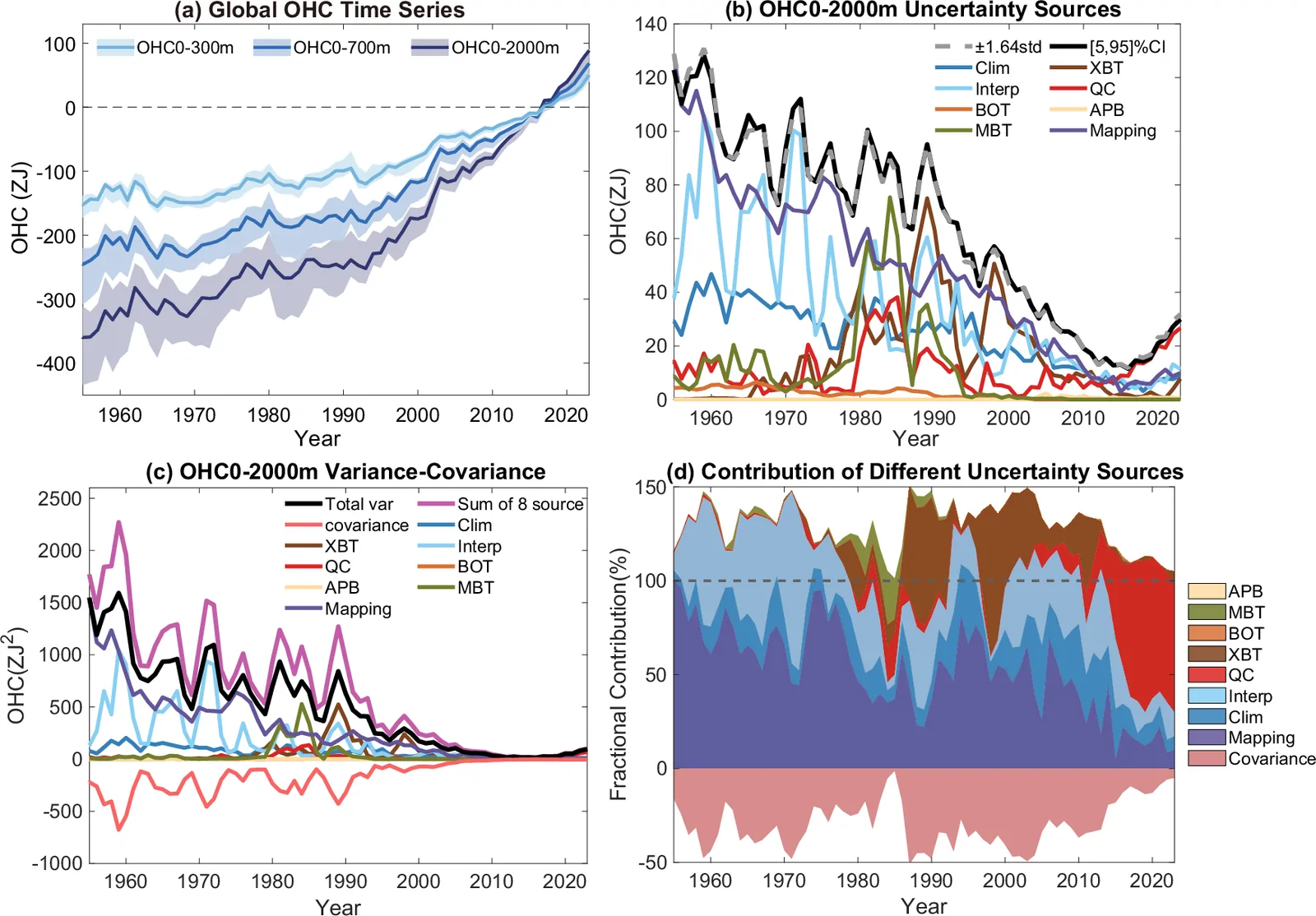
Global OHC (Ocean Heat Content—OHC) and its uncertainty from 1955 to 2023. **a** Global upper 300 m, 700 m and 2000 m OHC estimate by IAP (Institute of Atmospheric Physics—IAP) data (central estimate) and its uncertainty envelope (5%-95% quantile of the large ensemble computed on an annual basis), relative to a 2010-2023 baseline (Unit: ZJ); **b** OHC0-2000 m uncertainty from 8 major error contributions (colored lines) and the total uncertainty estimated from the large-ensemble (5%–95% quantile range estimated on an annual basis, solid black line), compared with the ±1.64 standard deviation range (dashed grey line, Unit: ZJ); **c** Global OHC0-2000m variance and covariance time series from 1955 to 2023 calculated by Eq.(1), including the variance of various error sources (colored lines), their sum (magenta line), the total variance of the large ensemble (black line), and the covariance term (pink line) (Unit: ZJ^2^); **d** Fractional contributions of different error sources (positive colored shadings) and covariance (negative pink shading) to the total variance of global OHC0–2000 m estimates. The sum of contributions from all error sources and the covariance equals 100. (Unit: %).

Global upper 2000 m OHC (OHC0-2000m) estimates suggest a robust increase since 1955 (449 [385, 519] ZJ, and 314 [281, 370] ZJ for 0–700 m; 468 [386, 535] ZJ for full-depth) with larger uncertainty in earlier periods revealed by the Large-Ensemble (Fig. 1a). The overall OHC0-2000m uncertainty, represented by the range of the [5%, 95%] quantile of the Large-Ensemble, is reduced from ~111 ZJ for 1955–1965 to ~19 ZJ for 2014–2023 for the annual mean OHC, indicating a nearly six-fold reduction in uncertainty and an increased precision in the OHC estimate.

The dramatic reduction of the uncertainty is attributed primarily to the decrease in mapping uncertainty (a combination of mapping/sampling and instrumental errors), which is progressively reduced from ~97 ZJ within 1955–1964 to ~7 ZJ for 2015–2023 (Fig. 1b, d; Supplementary Figs. 4, 6). The mapping uncertainty decrease is mainly linked to the substantial improvement of data coverage and sampling over time^28^, including the establishment of the Argo network since the beginning of this century which achieves a near-global open ocean coverage at upper ~2000 m^32^. The instrumental errors^18^ are much smaller than the mapping error.

Before ~1980, the vertical interpolation method was the second largest source of OHC0-2000m uncertainty, on average ~60 ZJ from 1955 to 1980 (Fig. 1b, c, d), associated with relatively lower vertical resolution of earlier temperature profile observations^24^, especially in the deep ocean below 700 m (Supplementary Fig. 4). There is a decadal-scale fluctuation of the vertical interpolation uncertainty, also mainly associated with the change of data resolution (Fig. 1c). However, the impacts of vertical resolution also depend on other factors such as spatial distribution; for instance, if a low-resolution profile is located in a data-sparse area, the impacts will be much bigger than when it is surrounded by many high-resolution data. The large vertical-sampling uncertainty is revealed by a notable difference between the PCHIP and RR approaches; the former yields larger OHC long-term trends than the RR method^24,25,33^. PCHIP considers the shape of the typical ocean temperature profiles and is considered to be more physically sound^25,33^.

The choice of climatology is the third largest uncertainty before ~1980, reducing from ~34 ZJ within 1955–1965 to ~6 ZJ for 2014–2023 (Fig. 1b). The climatology makes an impact because it is used to calculate the regional temperature anomalies that are input to the mapping procedure, and the impact of climatology is much larger in the deep ocean below 700 m, probably associated with less data there to estimate the climatology (Supplementary Fig. 4).

For the most recent period (after 2010), it is surprising that the total uncertainty increases from 2015 to 2023. Data QC has become the major source of error (~13 ZJ on average from 2010 to 2023), overwhelmingly dominating the OHC uncertainty (Fig. 1b). Specifically, the QC uncertainty is ~1.7 times larger than mapping. An additional test using removing one of the two QC systems suggests a substantial reduction of the uncertainty range (e.g., CODC-QC only ensemble spread is ~10 ZJ within 2015–2023, compared with total uncertainty range of ~19 ZJ) and a reduced increasing trend since ~2015, confirming that QC is mainly responsible for the recent uncertainty increase. An investigation indicates that the WOD local climatological range in its QC check, which is constructed by all historical data, mainly represents the historical ocean conditions and thus removes more positive temperature anomalies in the eddy-rich regions (such as Boundary currents and Antarctic Circumpolar Currents) than CODC^34^ (Supplementary Figs. 5, 6).

Unlike previous assessments where instrumental biases are the major error source^16,26^, this study finds a moderate contribution of four instrumental biases, implying a substantial reduction in uncertainty after bias correction (i.e., there is a better agreement among the three correction schemes, which leads to a smaller spread among corrected data, indicating biases can be well corrected). The OHC0-2000m uncertainty due to bias correction is notable from the late 1970s to ~2000, exhibiting peaks exceeding 70 ZJ in certain years and contributing an average of ~35 ZJ (XBT) and ~24 ZJ (MBT) over 1980–2000 (Fig. 1b, d). Although XBT data became a dominant data source from the late 1960s to 1980, the XBT data uncertainty is relatively small during 1960-1980 (~10 ZJ, Fig. 1b, d), indicating a consistent performance of the three XBT bias correction schemes^26^. Similarly, MBT bias correction uncertainty is only ~12 ZJ before 1980 (Fig. 1b, d), suggesting consistent corrections of MBT data by different schemes. In addition, APB bias correction uncertainty is <1 ZJ since ~1997, where APB data become available (Fig. 1b, d). The small APB bias uncertainty is because they are predominantly in the polar regions, and the impact on global OHC is not as large as other bias corrections (XBT/MBT). Moreover, the BOT bias correction is ~3 ZJ before 1990 and becomes near zero afterward because of the dismissal of BOT in recent decades (Fig. 1b, d).

**Fig. 2:**
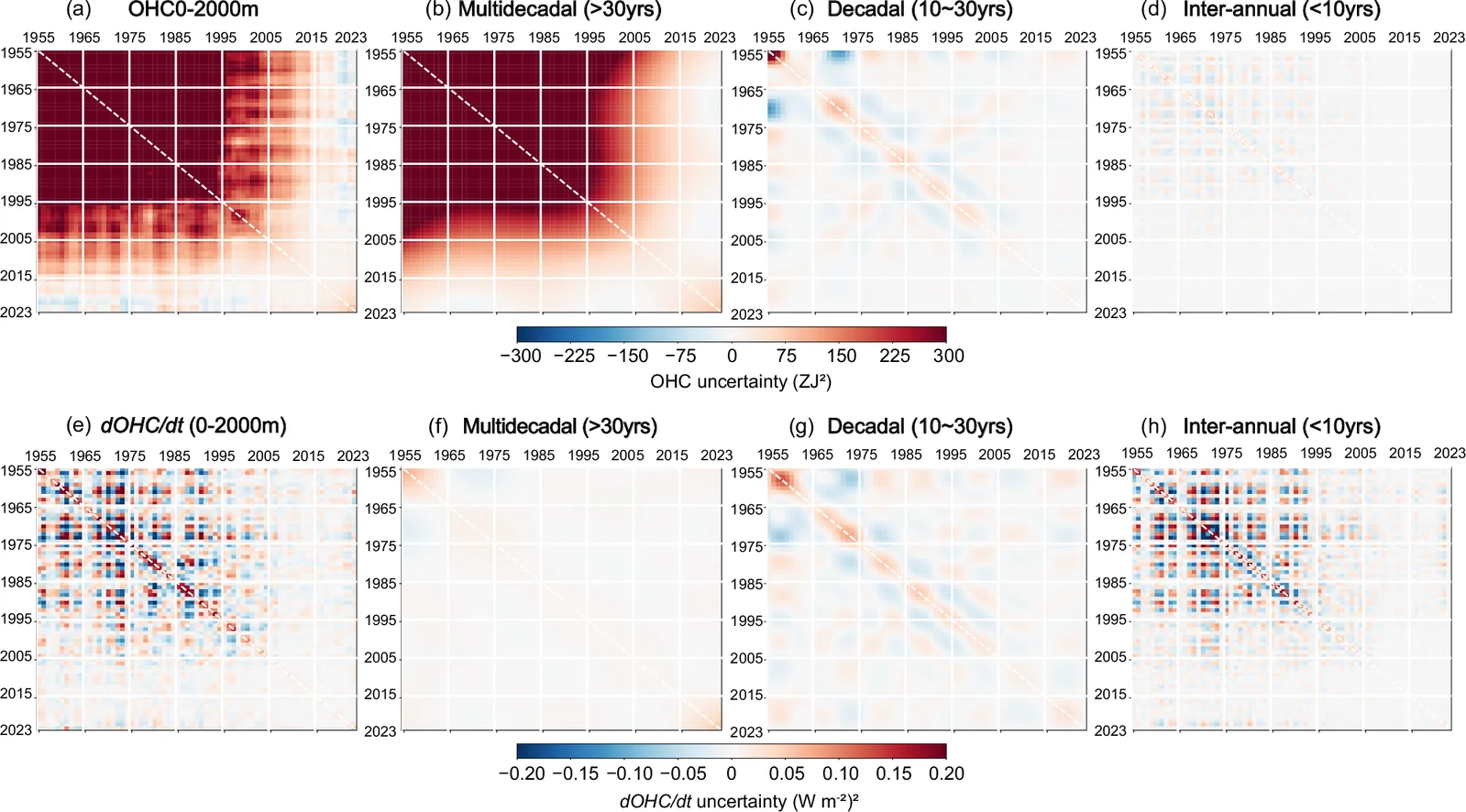
The variance-covariance matrix for global OHC (Ocean Heat Content—OHC) 0–2000 m (a–d) and *dOHC/dt* (e–h) uncertainty estimated by Large-Ensemble from 1955 to 2023. The variance–covariance matrix at different time scales is calculated: the raw variability is shown in (**a**, **e**); multidecadal variability calculated by ensemble members filtered by a 30-year low-pass filter (**b**, **f**); decadal variability with a 10–30-year band-pass filter (**c**, **g**), and inter-annual variability derived from a 10-year high-pass filter (**d**, **h**).

**Fig. 3:**
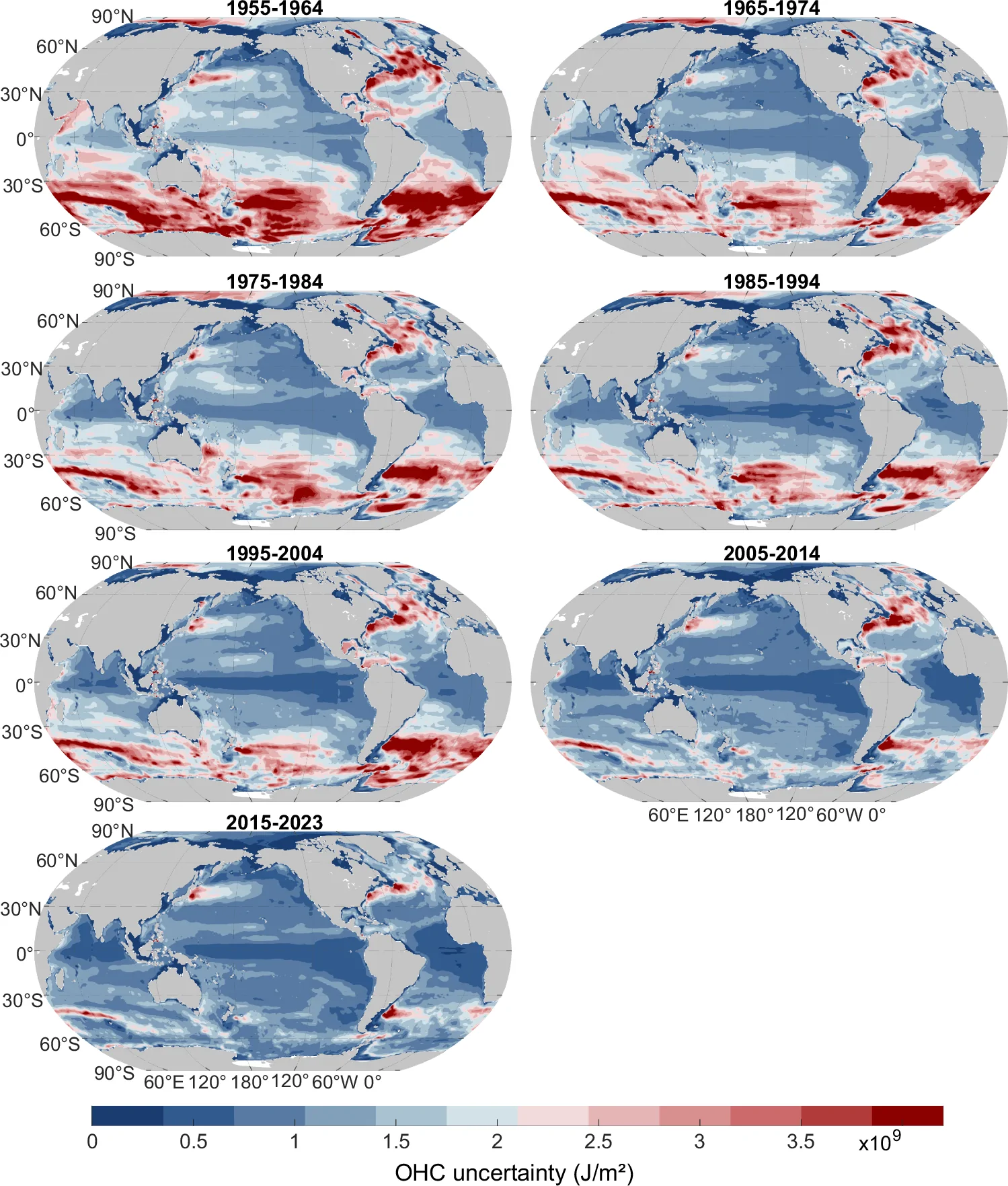
Regional total uncertainty (5–95% quantile range) for OHC (Ocean Heat Content—OHC) 0–2000 m derived by the large-ensemble approach in each decade since 1955. Unit: J/m^2^. Colored shading represents the magnitude of the total uncertainty.

**Fig. 4:**
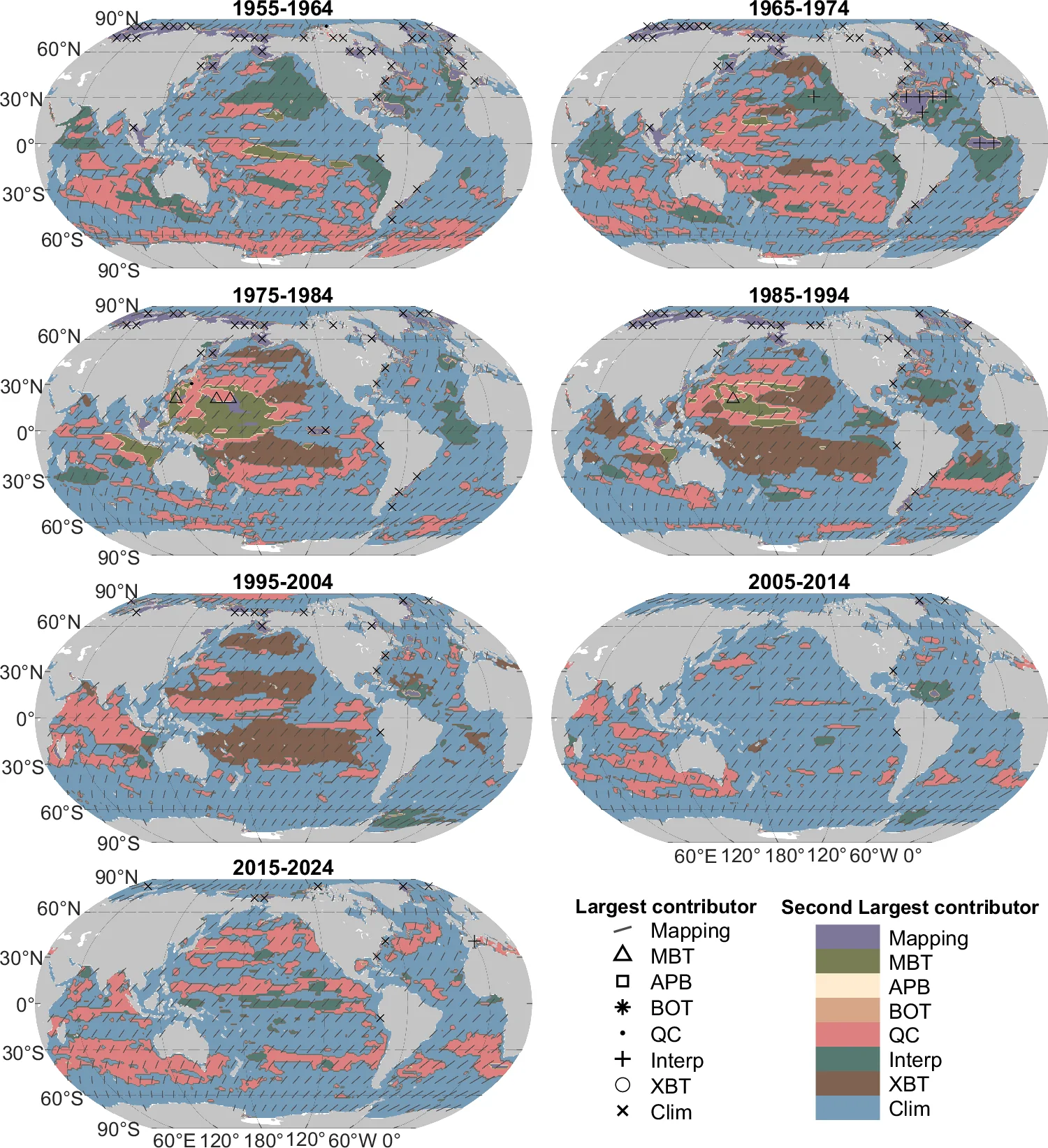
The first and second largest contributors of the regional OHC (Ocean Heat Content—OHC) 0–2000 estimates in each 1° by 1° grid for different decades. Colored shading indicates the second largest source of uncertainty, while symbols denote the largest contributor.

As the error sources are not independent, the sum of all uncertainties is larger than the total OHC0-2000m uncertainty (Fig. 1c). This is expected because each technique uses the output from the other procedures or provides an input to another procedure, thus linking error sources. Interestingly, the total covariance changed over time, being larger in the earlier periods (Fig. 1c, d). This effect is induced by the smaller number and less precise measurements in the earlier periods, in particular before the deployment of Argo floats. The variance–covariance matrix of the global OHC0-2000m change (Fig. 2a) shows substantial long-term covariances, which are dominated by multi-decadal scale covariances (>30 years) over decadal (10 – 30 years) and inter-annual scale covariances (<10 years) (Fig. 2), in particular before 2005 and the deployment of the Argo network. Hence the OHC errors are mainly manifested on decadal/multi-decadal scales. This large multidecadal covariance in global OHC is likely due to much less sampling before than after 2005 (in particular in regions far from trade routes like the Southern and the Arctic oceans or in regions with highly variable OHC as in western boundary currents, see Fig. 3). Less sampling led to notable errors in quality control, the climatology and also in mapping which are correlated in time (and eventually in space as well, see Fig. 4 and Supplementary Figs. 7-14). The sampling/mapping error (in particular below 700 m depth) before 2005 is also likely a contributor to these large multidecadal error covariances (Supplementary Fig. 4). The inter-annual scale covariances are larger before than after 2000 (Fig. 2d) probably for similar reasons. By contrast, the error covariance matrix for the derivative of OHC ($d{OHC}/{dt}$, ocean heat rate) is dominated by error covariances on interannual time scales. This is caused by the amplification of high-frequency errors/noise when doing the time derivative for the annual mean time series of OHC.

### Regional OHC uncertainty

The spatial maps of uncertainty indicate that uncertainty reduction has occurred in most ocean regions over the past six decades, with different patterns over time (Figs. 3, 4, Supplementary Figs. 7-14). In the earlier periods, the OHC0-2000m uncertainty distribution is characterized by larger errors in the Southern Hemisphere (2.4 × 10^9^ J m^-2^ and 2.0 × 10^9^ J m^-2^ on average from 1955–1964, 1965–1974, respectively, Fig. 3) than in the Northern Hemisphere (1.4 × 10^9^ J m^-2^ and 1.2 × 10^9^ J m^-2^ on average from 1955–1964, 1965–1974 respectively), primarily due to fewer observations and thus mapping uncertainty, Supplementary Fig. 7). Indeed, there are more cruises and human activities in the Northern Hemisphere. Hence, mapping is the largest source of uncertainty for almost all regions and across all decades (Fig. 4), so that sampling and associated mapping techniques remain the major limitation for a more accurate OHC estimate.

The choice of climatology was the second-largest source of error in at least half of the ocean area over the past 60 years (Fig. 4, Supplementary Fig. 8), owing to differences in regional anomaly calculations. The climatology-induced uncertainty can reach a maximum of > 1.3×10^9^ J m^-2^ in the eddy-rich regions. However, these regional climatology-induced errors do not seem to contribute substantially to global mean and trend estimates (see other sections of this study), indicating regional compensation.

The uncertainty in mid-latitude OHC0-2000m has been reduced since the mid-1960s due to the widespread use of XBT data (Fig. 3). Meanwhile, XBT and MBT bias corrections became the second-largest source of uncertainty in the mid-latitude Pacific Ocean from 1965 to 2004 (Fig. 4, Supplementary Figs. 9–10). The MBT/XBT bias correction uncertainty is more important in the Pacific Ocean than the Atlantic Ocean because there is also a large amount of more accurate CTD data in the Atlantic, while MBT/XBT dominates the Pacific Ocean observing system during these periods^28,32^ (Fig. 4, Supplementary Figs. 9–10). BOT bias is critical for the North Pacific and Atlantic Oceans before the 1990s (Supplementary Fig. 11).

The tropical OHC uncertainty has been reduced since the 1980s (Fig. 3), largely due to the establishment of the tropical moored buoy system designed to track El Niño. Indeed, the tropical ocean within 10^o^S ~ 10^o^N is the region with the smallest OHC uncertainty in the recent decade (2015–2023, <0.3×10^9^ J m^-2^), highlighting the importance of maintaining the tropical observing system.

The vertical interpolation has become the second largest source of uncertainty in the North Pacific from 1955 to 1974, tropical and subtropical Atlantic Ocean from 1955 to 2014 (up to 1 × 10^9^ J m^-2^, Supplementary Fig. 12), owing to a significant amount of low-resolution data^24^. Vertical interpolation is also critical in ACC and boundary current regions, where strong vertical gradients are present.

**Fig. 5:**
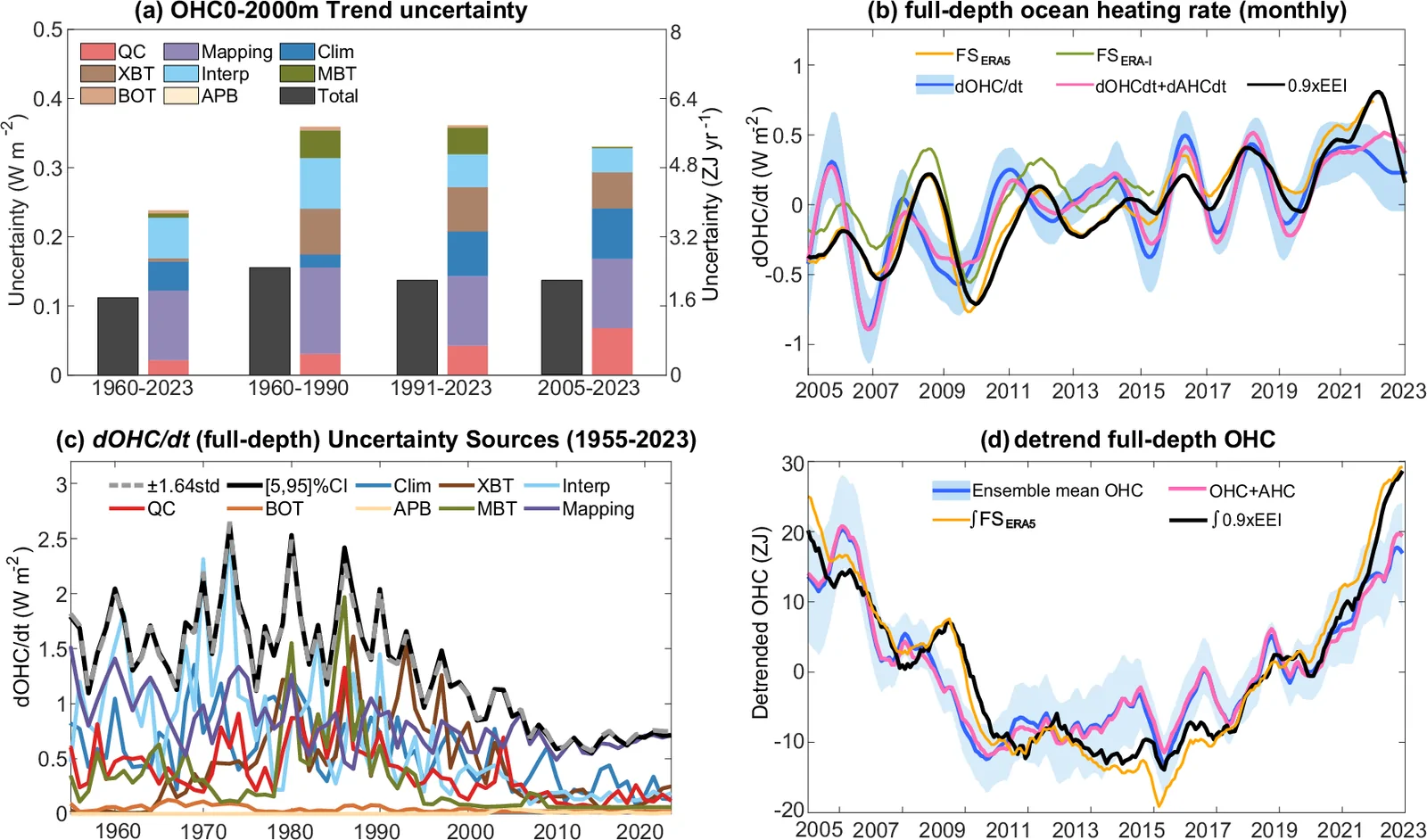
Uncertainty of the OHC (Ocean Heat Content—OHC) trend and variability. **a** Uncertainty of the OHC0-2000 trend over different periods: 1960–2023, 1960–1990, 1991–2023, and 2005–2023, compared with the sum of uncertainties from different error sources. The [5%, 95%] ranges are shown (unit: W m^-2^ and ZJ yr^-1^). **b** Ocean heating rate estimated by full-depth $d{OHC}/{dt}$, Wm^-2^, blue shading for [5%, 95%] range with blue line for the ensemble mean estimate compared with the 0.9 × CERES-EEI (Clouds and the Earth’s Radiant Energy System Earth’s Energy Imbalance—CERES-EEI) data, two oceanic FS (Surface Net Heat Flux) and the sum of $d{AE}/{dt}$ (Atmospheric Energy—AE) and $d{OHC}/{dt}$, which are mean-aligned to $d{OHC}/{dt}$ by removing the mean difference over 2005–2023. Earth’s surface area (5.1 × 10^14^ m^2^) is used to calculate heating rates. **c** Uncertainty (5% ~ 95 range) of the annual full-depth OHC rate ($d{OHC}/{dt}$). The contributions of different error groups are shown in different colors. Unit: W m^-2^. $d{OHC}/{dt}$ is calculated by the difference between the average values of the preceding and following twelve months, and the resultant time series is smoothed by a 15-month running mean to reduce the month-to-month noise. **d** Same as (**b**) but for the detrended full-depth OHC, the detrended sum of full-depth OHC and AHC (Atmospheric Heat Content—AHC), time-integrated CERES observations at TOA (Top of the Atmosphere—TOA) (detrended), and time-integrated global ocean FS for 2005–2023 period (detrended).

In earlier periods, QC impacts (Figs. 3, 4) were primarily observed in the middle parts of the Pacific and Indian Oceans and the Southern Ocean (before 2004), where XBT, MBT, and BOT data dominated and often contained more erroneous data than other instruments, such as CTD and moored observations. Since ~2005, QC impacts are substantial in the eddy-rich regions (Fig. 5) where the handling of the large anomalies associated with eddies becomes critical (Supplementary Figs. 5, 6, 13).

### Global OHC trends and uncertainty

OHC trends and variability uncertainty are separated from the annual mean estimates in previous sections, because the precision of the trend and variability estimates are impacted by both annual OHC uncertainty and its time variation and time correlation. The trend of OHC0-2000m from 1960 to 2023 is estimated to be 6.4 [5.5, 7.4] ZJ yr^-1^ (equivalent to 0.40 [0.34, 0.46] Wm^-2^ over the global surface area), with the error range represented by [5%, 95%] quantile of all ensemble members (Fig. 5a). The first and second sources of error in 1960–2023 OHC0-2000m trend are mapping (1.6 ZJ yr^-1^, hereinafter calculated by 2 × 1.64-sigma based on ensemble members fixing other techniques) and vertical interpolation (1.0 ZJ yr^-1^), respectively, followed by climatology choice (0.7 ZJ yr^-1^) and QC (0.3 ZJ yr^-1^). Again, the sum of errors is two times larger than the total trend uncertainty, implying strong error covariance impacts on trend estimate (Fig. 5a).

**Fig. 6:**
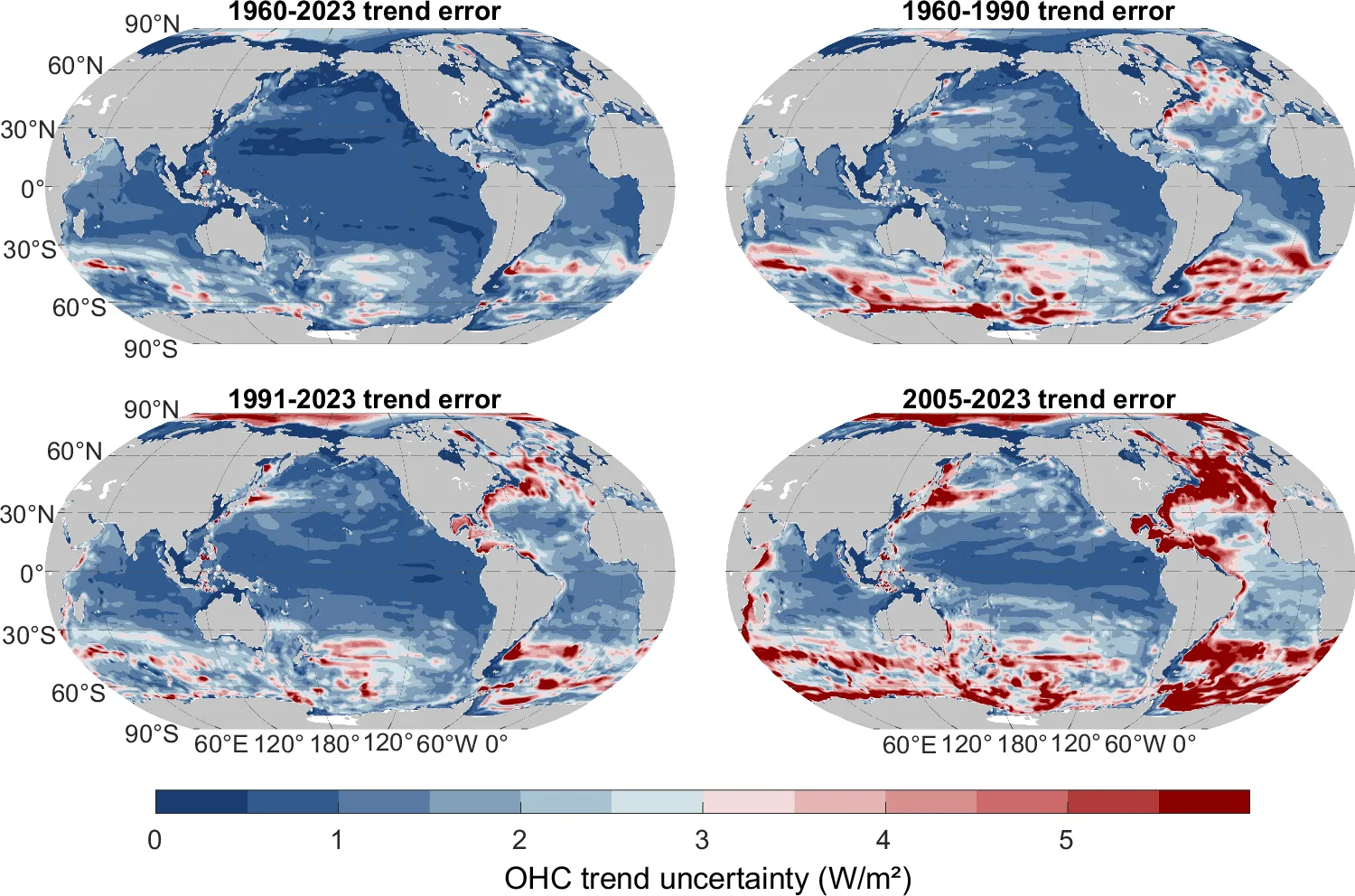
OHC (Ocean Heat Content—OHC) 0-2000 m trend uncertainty over different time periods. Unit: W/m^-2^. The time periods are 1960–2023, 1960–1990, 1991–2023, and 2005–2023. Colored shading represents the magnitude of the trend error.

The OHC0-2000m trend has increased over time: 2.8 [2.0, 4.3] ZJ yr^-1^ (equivalent to 0.18 [0.12, 0.27] Wm^-2^ over the global surface area) from 1960 to 1990, 9.9 [8.5, 10.6] ZJ yr^-1^ (0.61 [0.53, 0.66] Wm^-2^) from 1991 to 2023, and 10.9 [9.2, 11.4] ZJ yr^-1^ (0.68 [0.57, 0.71] Wm^-2^) from 2005 to 2023 (Fig. 5a). As their error ranges do not overlap, the rate changes are statistically significant, increasing the robustness of the multidecadal ocean warming acceleration^3,6–8^. For all time periods, the regional OHC0-2000m trend uncertainties are larger in the Southern Ocean (78-30S), western Indian, Arctic, and boundary currents regions than in other regions (Fig. 6), probably associated with higher variability in these regions and also greater noise. However, the primary contributions of error sources vary across different periods. For instance, the mapping is more important for 1960–1990 than 1991–2023 because of the improved sampling in recent decades (Fig. 5a). Similarly for the vertical interpolation impact, because of the increased vertical resolution of ocean profile data over time (Fig. 5a). Since 2005, QC has substantially impacted the trends, suggesting the need to address better eddy-rich regions where anomalies are large, often associated with significant thermocline variations in eddies (Fig. 5a).

### Ocean heating rate

The centered difference of the OHC time series ($d{OHC}/{dt}$, full-depth) is often used to calculate ocean and Earth’s heating rate (Fig. 5b). But there is substantial month-to-month variability of the ocean heating rate^8,35^. Because the first-time derivative can amplify noise/errors in time series, the uncertainty can be substantial and is assessed in this section. With this approach, the monthly ocean heating rate uncertainty (5–95% quartile range of annual $d{OHC}/{dt}$) is estimated to be ~1.54 Wm^-2^ from 1955 to 1975, which is reduced by over 2-fold in this century, which is 0.67 Wm^-2^ from 2005 to 2023 (Fig. 5c). Vertical interpolation is the primary source of error that generates uncertainty in the inter-annual scale OHC change before ~1975 (Fig. 5c), as the vertical sampling of ocean temperature profiles exhibits substantial year-to-year differences. The second largest error source is mapping, followed by QC and XBT bias corrections (Fig. 5c). In the recent decade, mapping is the dominant error source, followed by the choice of climatology (Fig. 5c). This big impact of climatology is a surprise, and it seems to be because of the impacts on the eddy-rich regions, probably associated with the poor definition of a mean state in these regions. Overall, trend errors are larger in recent decades, owing to the short record length as well as larger underlying trends and variability (Fig. 6).

Ocean heating dominates the EEI from inter-annual to multi-decadal time scales^4,8,14,36^, thus *dOHC/dt* is compared with 90% of CERES-EEI and FS derived from CERES and atmospheric reanalysis (Fig. 5b). The *dOHC/dt* is correlated with CERES-EEI by 0.61 from 2005 to 2023, suggesting a reasonably good closure of the global energy budget. From 2005–2023, satellite CERES (FS) suggests an ocean heating rate of 0.48 ± 0.13 Wm^-2^ dec^-1^ (0.38 ± 0.28 Wm^-2^ dec^-1^, mean of ERA5 and ERA-Interim), slightly stronger than the estimated $d{OHC}/{dt}$ trend of 0.34 [0.19, 0.46] Wm^-2^ dec^-1^ based on IAP central estimate (Fig. 5b, c). The IAP central estimate is closer to the upper end of the ensemble members because it used CODC-QC, which yields stronger trends than the results using WOD-QC. Adding atmospheric heat content^37^ into $d{OHC}/{dt}$ further increases the correlation with CERES-EEI, which becomes 0.7, indicating a substantial contribution from the atmosphere^38^. However, CERES also has sampling, diurnal cycle and satellite/instrument changes uncertainties. Nevertheless, the year-to-year variability of OHC and EEI requires further investigation^39^. Furthermore, as calculating time derivatives exacerbates noise, we integrate EEI and FS time series and compared the detrended $\int {EEI}$ and $\int {Fs}$ data with detrended OHC (Fig. 5d). This comparison shows better consistency. This indicates that the data processing techniques (in particular, derivation techniques) also matter for an OHC and EEI comparison^40^.

## Discussion

This study carried out a large-ensemble approach to quantify the OHC uncertainty from all major error sources. The methods used techniques developed by many international groups, thus reflecting the international community’s progress over the past decade. The results indicate a substantial reduction in OHC estimate error since the 1950s, from ~111 ZJ from 1955–1965 to ~19 ZJ from 2015 to 2023. The uncertainty reduction seems obvious because of the establishment of Argo and other networks in this century^41^. However, given the caveat that WOD-QC removes more realistic positive anomalies in eddy-rich regions^42^, the QC uncertainty is likely to be overestimated in recent decades. If taken CODC-QC as a single choice of QC, the OHC uncertainty since 2015 is ~10 ZJ on average. Long-term increase of ocean warming rate (OHC0-2000m) is robust, indicating a multi-decadal scale ocean warming acceleration.

However, the annual ocean heating rate is more uncertain due to data and methodological uncertainties, including those associated with the calculation of the first derivative. From 2005–2023, the annual full-depth $d{OHC}/{dt}$ time series shows a trend of 0.34 [0.19, 0.46] Wm^-2^ dec^-1^ (5%-95% uncertainty range calculated by the Large-Ensemble), smaller than satellite CERES estimate during the same period (0.48 ± 0.13 Wm^-2^ dec^-1^), but the uncertainty range overlaps. All ocean regions show a substantial reduction in OHC uncertainty over the global ocean in the past half-century, especially in the Southern Hemisphere, owing to improved data coverage and quality. In the recent decade, eddy-rich regions have shown greater OHC uncertainty than other regions, highlighting critical areas for improving OHC and its trend estimates. Changes in error sources over time demonstrate how the dominant sources of uncertainty evolve and depend on changes in observational platforms and methodologies, and underscore the importance of properly accounting for both individual error sources and their correlations.

The strength of the Large-Ensemble approach lies in its ability to revisit and integrate all known techniques to examine the spread, given our current understanding of the problem. The covariance among various error sources can also be quantified. However, the weakness of ensemble approaches is that they cannot investigate the “unknown unknowns” (for instance, the not-yet-identified instrumental biases). In this regard, the consequence is that the ensemble approach might provide a lower bound for the real uncertainty. Therefore, when any new error sources are discovered, they should be incorporated into this effort. Even if systematic errors and unknown unknowns are omitted, their impact is likely small on the variability and the trend of the OHC, as it shows reasonable agreement, mostly within error bars, with an independent estimate based on CERES and atmosphere reanalysis. An additional caveat of this study is that the mapping uncertainty is quantified with the IAP ensemble approach in a different framework than other sources. We acknowledged the challenge of quantifying sampling/mapping uncertainty: the truth in data gaps will not be known, and a mapping approach is used to fill the gaps.

Besides, the uncertainty quantified in this study only presents an in-situ observation-based OHC estimate^3^, where a similar protocol is used for various groups^31,43,44^. However, there are other approaches for OHC estimation, such as an indirect approach based on geodetic techniques using satellite altimetry and gravity to derive OHC^15,41,45^ or hybrid approaches using both in-situ profiles and geodetic techniques. The uncertainty budget associated with these indirect approaches is different and requires separate treatment.

A closer look at the uncertainty in the current decade has critical implications for improving the current GOOS and OHC/EEI estimate:

1. The eddy-rich regions, including boundary currents and ACC regions, should be better dealt with, because they contribute much larger uncertainty than the other regions (Figs. 3, 4, 6). Critical improvements include increasing data coverage (improving data sampling), mapping technique (including improving resolution to resolve meso-scale eddies), improving climatology and QC procedure in these regions, and also utilizing other sources of information, such as altimetry, and other satellite observations^46,47^.
2. The global data coverage should be increased over the global ocean where practicable, as mapping is still the dominant uncertainty source in most of the ocean area (Figs. 3, 4, 6). Specifically, the Global Argo network contains ~4000 floats with an average resolution of 3° by 3° and 1 month. This network should be extended horizontally and vertically to reduce mapping uncertainty^32^. Besides Argo, ship-based observations, Moorings, Animal Borne Ocean Sensors also needs to be sustained and expanded to fill the critical gaps and build a resilient GOOS^48^.
3. The data quality and QC procedure should be further improved (Figs. 1, 5), including a way to investigate why QC leads to recent substantial uncertainty, what the best QC practices are, and how to remove the systematic errors in ocean observations. The exploration could also extend to the ocean conditions under extreme events such as tropical cyclones and marine heat waves, where anomalous large but realistic values occur.
4. Closure of the Earth’s energy budget using CERES radiation, land, cryosphere and atmosphere data would be critical to test the validity of the GOOS and will provide clues for its improvements. The closure of sea level budget provides an important check for OHC given that the altimetry and GRACE observations provide reliable changes in global mean sea level and its mass component^49^.

Comparing OHC with EEI and surface flux data shows notable differences on the inter-annual scale, although the warming acceleration agrees between these 2 totally independent estimates meaning it is robust. Integrating ocean in situ ocean data with other observations, such as altimetry, satellite gravity data, CERES, and ocean color data using data merging techniques, data assimilation, and Artificial Intelligence methods, seems a promising way forward. Furthermore, as the atmosphere and ocean circulations move heat around, the regional pattern of OHC is distinct from EEI. The OHC and EEI can be better understood and reconciled through an integrated understanding of Earth’s energy budget and there is a heat redistribution between ocean and atmosphere, land, and cryosphere^50,51^.

## Methods

### Data

The main data sources come from the in-situ temperature profile data in the World Ocean Database (WOD) downloaded in August 2024, which is then organized in the CODC database after adding more in situ observations from other data sources^52^. Measures from all instruments are used, including Conductivity/Temperature/Depth (CTD), XBT, MBT, Argo, BOT, moorings (MRB), gliders (GLD), APB, and others. The OHC calculation procedure follows the IAP group system^53^, which is similar to those used elsewhere^43,44,54^.

TOA net radiative flux timeseries data is from the CERES Energy Balanced and Filled (EBAF) Ed4.2 product^55^. CERES instruments have provided continuous broadband radiation measurements since March 2000. The CERES EBAF Ed4.2 record delivers consistent monthly mean shortwave, longwave, and net radiative fluxes at the TOA, and is widely regarded as the reference dataset for studies of Earth’s radiation budget. In our comparisons with $d{OHC}/{dt}$, we consider the period from January 2005 to December 2023. However, it contains noise on monthly time scales owing to limited sampling of the diurnal cycle^56^.

The surface net heat flux (*FS*) products can be derived from observations of net energy budget observations at the TOA, the atmospheric energy storage and the convergence of the vertically-integrated atmospheric energy fluxes from different atmospheric reanalysis products^57–59^. Two *FS* products are used in this study: The first *FS* product is obtained by combining DEEPC v5.0 TOA fluxes^59^ for 1985–2000, adjusted to match CERES EBAF Ed4.2 (Ref. ^55^) by adding the mean annual cycle difference between CERES EBAF Ed4.1 and Ed4.2 (estimated over 2001–2018), and CERES EBAF Ed4.2 thereafter, combined with ERA5 (Ref. ^37^) for January 1985- December 2023. The second *FS* product is obtained by combining CERES v4.0 TOA radiation and ERA-Interim^60^ for March 2000-December 2016 (ref. ^61^).

The atmospheric heat content (AHC) data is computed based on data from the fifth generation of European ReAnalysis (ERA5, Ref. ^37^) monthly single-level reanalysis produced by the European Centre for Medium-Range Weather Forecasts (ECMWF). In this study, we use the total column vertically integrated total energy, which represents the vertically integrated sum of internal, potential, kinetic, and latent energy over the full atmospheric column.

### Large ensemble construction

**Fig. 7:**
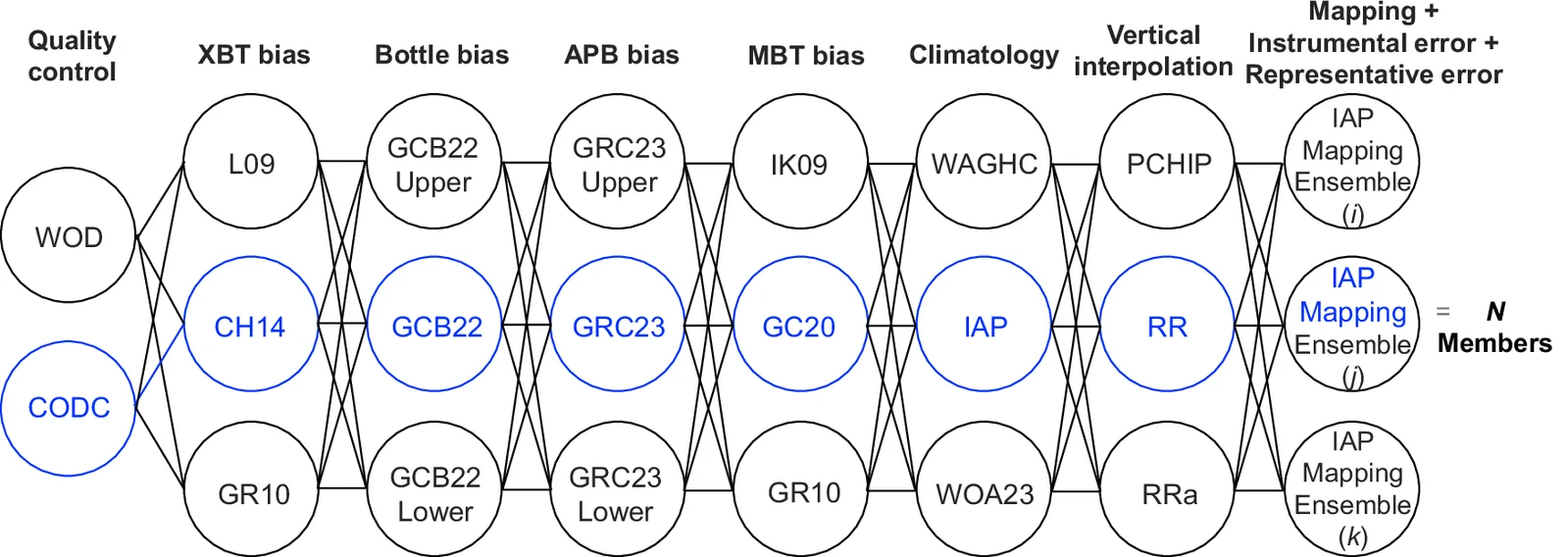
Design of the observational large-ensemble approach. The techniques with blue colors are used to derive the IAP (Institute of Atmospheric Physics—IAP) central estimate, as they are assessed as the most up-to-date or best choices.

Many steps must be used to calculate OHC from in situ observations, and all contribute to the uncertainty of the final OHC estimate. On this basis, we developed a large-ensemble approach to quantify uncertainty. For each given step, the three most up-to-date techniques (developed by the many international ocean data groups over the world) are used to give three different estimates whose standard deviation serves as the error estimate associated with the given step (except for mapping, as discussed later), as outlined below. Three techniques are used because 1) the computational power is limited, and 2) only the state-of-the-art knowledge is considered to avoid overestimating the uncertainty. An example is XBT bias correction schemes; there are more than 10 correction schemes available, many of which have been investigated in previous efforts^16,26^. But recent investigations^20,62^ have revealed caveats for many correction schemes. For example, some correction schemes only considered part of the XBT biases. The choices of each error source at each step are briefly discussed below (Fig. 7), and one of them (Fig. 7, marked as blue texts) is used to derive the IAP central estimate, which is assessed as the state-of-the-art estimate for the IAP dataset (see Ref. ^53^):

1. QC: WOD^42^ and Chinese Academy of Sciences Ocean Data Center (CODC)^34^. The two systems use very different QC checks and represent different strategies for QC of ocean data. One of the major differences is the local climatology range change. While WOD-QC uses a static climatological range constructed from all historical data to filter out outliers, CODC-QC uses a time-varying range which considers the long-term trends of ocean changes^63^ that are non-trivial. For QC, only two systems are used because of the limited access to other systems but the two are representative.
2. Three best XBT correction schemes are used: Cheng et al. (Ref. ^63^., CH14); Levitus et al. (Ref. ^31^., L09) and Gouretski and Reseghetti (Ref. ^64^., GR10) following the evaluation in previous studies^62^ and community-recommendations^20^. CH14 introduces a time- (annual mean), probe-type and temperature- dependent correction scheme, while L09 is a global mean time-varying scheme and GR10 considers the bias dependence on probe-type and time.
3. Bottle bias correction: the central estimate and an upper and lower bound of the correction developed in Gouretski et al. (ref., ^22^ GCB22). The upper and lower bounds represent conservative and aggressive estimates of the bottle bias.
4. APB bias correction: the central estimate, an upper and lower bound of the correction are used based on Gouretski et al. (Ref. ^23^., GRC23). Similar to bottle bias correction, GRC23 is the only correction scheme for APB bias, and the upper and lower bounds represent a conservative and aggressive estimate of the correction.
5. MBT bias correction: the central estimate is Gouretski and Cheng^21^, with two additional approaches: Gouretski and Reseghetti (ref., ^64^ GR10) and Ishii et al. (ref., ^65^ IK09). Another MBT bias correction scheme is available^66^ but is not used here as an assessment^63^ indicates a substantial suspicious deviation from the other schemes.
6. Choice of climatology: Three different methods of computing the climatology are used namely the World Ocean Atlas 2023 method (ref., ^54^ WOA), the Institute of Atmospheric Physics method (ref., ^53^ IAP); World Ocean Circulation Experiment-Argo Global Hydrographic Climatology method (ref. ^67^, WAGHC).
7. Vertical interpolation: Three methods are used namely: the Reiniger and Ross method(Ref. ^68^., RR), the Piecewise Cubic Hermite Interpolating Polynomial (ref., ^33^ PCHIP) applied to the temperature profiles, and the RR approach applied to temperature anomaly profiles after subtracting IAP climatology.
8. Mapping and instrumental error: only IAP mapping^69,70^ was used because the codes of other approaches are not publicly available. But IAP mapping can provide a reasonable estimate of mapping uncertainty, because it is an Ensemble Optimal Interpolation (EnOI) approach with the CMIP5 multi-model ensemble (30 models) as *prior* to represent our current understanding of the ocean changes (the larger spread in models, the poorer understanding of physical changes, especially in regions with large data gaps where there is no observational information to estimate uncertainty). The observation accuracy set by IQuOD^18^ is used to constrain the model spread and derive the final ensemble of the analysis field. Thus, the more data and the higher quality, the smaller the ensemble spread in the final analysis, representing smaller uncertainty in reconstruction. Besides, in this approach, the instrumental, sampling, and mapping uncertainties are combined and are represented by the ensemble spread of the derived analysis field. An alternative approach to assess sampling/mapping errors is to resample the data in data-rich periods according to historical observation masks and assess the reconstruction error^69–71^, which provides reasonably consistent uncertainty estimates compared with the EnOI approach.

As introduced in mapping and instrumental error analysis, the mapping uncertainty (defined by the EnOI approach) is treated differently from the other error sources (defined as the differences between different techniques). We merged the mapping uncertainty with the other sources of error by randomly selecting one ensemble member derived from the EnOI ensemble (see Fig. 7).

Ideally, all combinations of error sources (first seven in Fig. 7) yield a large ensemble of 2*3^7^ = 4374. But in practice, there is limited computational power, and calculations show that the ensemble spread and the 10% quantile actually stabilize after only a few hundred members (Supplementary Fig. 1), meaning not all the 4374 members need to be computed to derive the actual spread of the ensemble and the estimate of the total uncertainty. We eventually made 908 ensemble members to satisfactorily derive the uncertainty and error covariances (Supplementary Fig. 2).

### Uncertainty quantification

Throughout the paper, the overall uncertainty is defined as the [5%, 95%] quantile range among ensemble members and is close to that obtained if the distribution was assumed as gaussian (see Supplementary Fig. 3), meaning the error distribution is likely not far from a gaussian distribution, as expected. For each error source, as only three different choices are made, the quantile is not feasible; instead, the uncertainty is represented by the 1.64 standard deviation (approximately equal to [5%, 95%] range assuming a gaussian distribution), calculated by the mean variance of the 2 or 3 choices of techniques with other error technical choices fixed.

The error covariance is inferred from the differences among the variance of all ensemble members and the sum of all variances of individual error sources:

1 $${{cov}}\left(e1,\ldots,e8\right)={Var}\left(e1,\ldots,e8\right)-[{Var}\left(e1\right)+{Var}\left(e2\right)+\ldots+{Var}\left(e8\right)]$$

Throughout this study, the “covariance” includes both linear and nonlinear relationships among variables. In addition, we derive the variance-covariance matrix for global OHC0-2000m time series using the Large-Ensemble to describe the time correlation in errors^72^. The ensemble OHC0-2000m time series (${{TS}}_{i}$) are directly used to calculate the time-covariance between each time series as follows:

2 $$\sum {OHC}\left(i,j\right)={{cov}}\left({{TS}}_{i},{{TS}}_{j}\right)=E\left({{TS}}_{i}-E\left({{TS}}_{i}\right)\right)\left({{TS}}_{j}-E\left({{TS}}_{j}\right)\right)$$

where E is the mean operator. This approach is reliable when the ensemble is large enough to describe the dispersion between ensemble members adequately, as is the case with 908 members.

For each OHC member, $d{OHC}/{dt}$ is calculated by using the central derivative of the annual (Jan.-Dec. average) OHC time series, and the long-term OHC trend is calculated by a LOWESS approach^73^. Then the uncertainty range is derived through the results of all ensemble members, through which the error covariance can be accounted for. An alternative approach using the variance-covariance matrix^72^ yields very similar results (Supplementary Table 1).

## Supplementary information

Supplementary Information

Transparent Peer Review file

## Data Availability

All the data generated by this study is available in http://www.ocean.iap.ac.cn/ftp/ensemble/. Data used to generate all analyses and figures in this study are also available in https://zenodo.org/records/21376891.

## Code availability

Codes used to generate all analyses and figures in this study are available in http://www.ocean.iap.ac.cn/ftp/ensemble/code/ and https://zenodo.org/records/21376891.
