# Supplementary material for: Six-fold reduction in ocean heat content estimate uncertainty since 1960
Source: Nat Commun. 2026 Aug 29;17:10317. doi: 10.1038/s41467-026-76436-0 (PMC13620135; doi:10.1038/s41467-026-76436-0)
Supplement: Supplementary file 1 [file 41467_2026_76436_MOESM1_ESM.pdf]

Supplementary Information for

Six-fold reduction in ocean heat content estimate uncertainty since 1960

Huifeng Yuan[1,2,3], Lijing Cheng[1,3*], Yuying Pan[1], Bin Zhang[4,5], Benoit Meyssignac[6], Kevin E. Trenberth[7,8], Yujing Zhu[1], Xinyi Song[1], Huayi Zheng[1], Senliang Bao[9], Juan Du[3,10], Jiang Zhu[1,3], Zhong Jin[2], Xuebin Chi[2], Jinrong Jiang[2], Rongwang Zhang[11], Yuan Tian[2], Ning Liu[2]

[1] State Key Laboratory of Earth System Numerical Modeling and Application, Institute of Atmospheric Physics, Chinese Academy of Sciences, Beijing, China, 100029

[2] Computer Network Information Center, Chinese Academy of Sciences, Beijing, 100083

[3] University of Chinese Academy of Sciences, Beijing, China, 100049

[4] Institute of Oceanology, Chinese Academy of Sciences, Qingdao, China, 266071

[5] Oceanographic Data Center, Chinese Academy of Sciences, Qingdao, China, 266071

[6] Université de Toulouse, CNES, CNRS, IRD, UT3, LEGOS, Toulouse, France

[7] National Center for Atmospheric Research, Boulder, CO, USA

[8] Department of Physics, University of Auckland, Auckland, New Zealand

[9] College of Meteorology and Oceanography, National University of Defense Technology, Changsha, China, 410073

[10] International Center for Climate and Environment Sciences, Institute of Atmospheric Physics, Chinese Academy of Sciences, Beijing, China, 100029

[11] South China Sea Institute of Oceanology, Chinese Academy of Sciences, Guangzhou, China, 510301

* Corresponding author: Lijing Cheng: chenglij@mail.iap.ac.cn

Content

Supplementary Table 1
Supplementary Figures 1-14

Supplementary Table 1. Trend uncertainty (5%-95% confidence interval range) calculated by using two approaches (Unit: ZJ yr^{-1}). Gray-shaded rows show results based on the LOWESS approach [1], whereas unshaded rows are derived from the Matrix method[2].

	1960-2023	2005-2023	1960-1990	1991-2023
Climatology (Matrix)	0.67	1.32	0.47	1.16
Climatology (LOWESS)	0.68	1.17	0.30	1.04
XBT correction (Matrix)	0.15	0.8	1.94	0.86
XBT correction (LOWESS)	0.07	0.85	1.07	1.04
Interpolation (Matrix)	1.09	0.48	1.33	0.71
Interpolation (LOWESS)	0.96	0.55	1.21	0.78
Quality Control (Matrix)	0.24	1.03	0.59	0.87
Quality Control (LOWESS)	0.35	1.08	0.48	0.68
BOT correction (Matrix)	0.09	0.00	0.11	0.03
BOT correction (LOWESS)	0.07	0.01	0.09	0.05
APB correction (Matrix)	0.02	0.04	0.0	0.02
APB correction (LOWESS)	0.01	0.01	0.00	0.02
MBT correction (Matrix)	0.3	0.01	1.14	0.17
MBT correction (LOWESS)	0.10	0.03	0.64	0.62
Mapping method (Matrix)	1.45	1.41	1.46	1.81
Mapping method (LOWESS)	1.63	1.62	2.01	1.64
ALL (Matrix)	1.82	2.16	2.66	2.28
ALL (LOWESS)	1.83	2.18	2.29	2.07

Supplementary figures

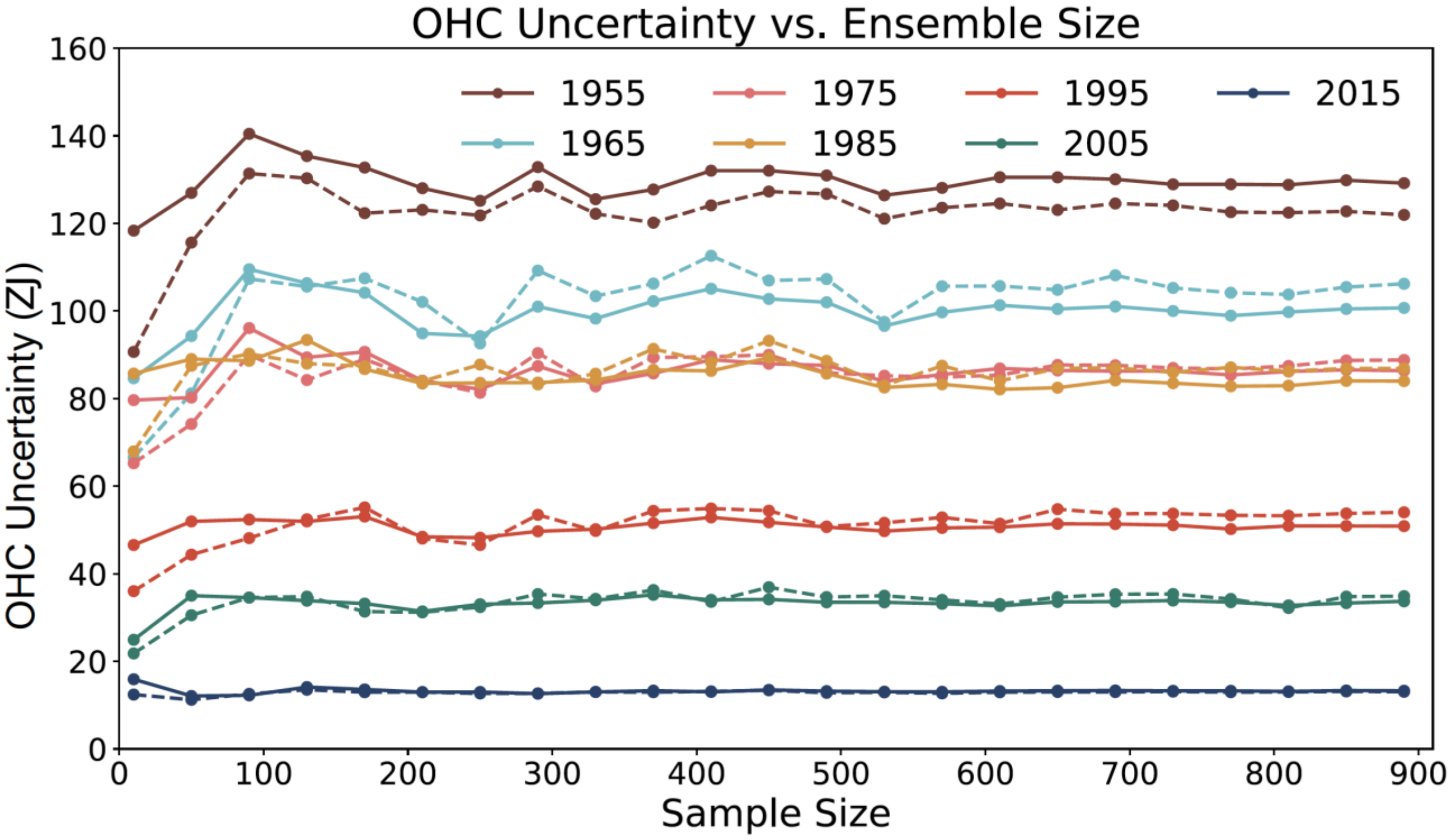

Supplementary Fig. 1. **Global OHC (Ocean Heat Content - OHC) 0-2000m shown as the 5-95% percentile error range (dashed lines) and twice 1.64 times the standard deviation (solid lines) estimates as functions of ensemble sample size for several years**.

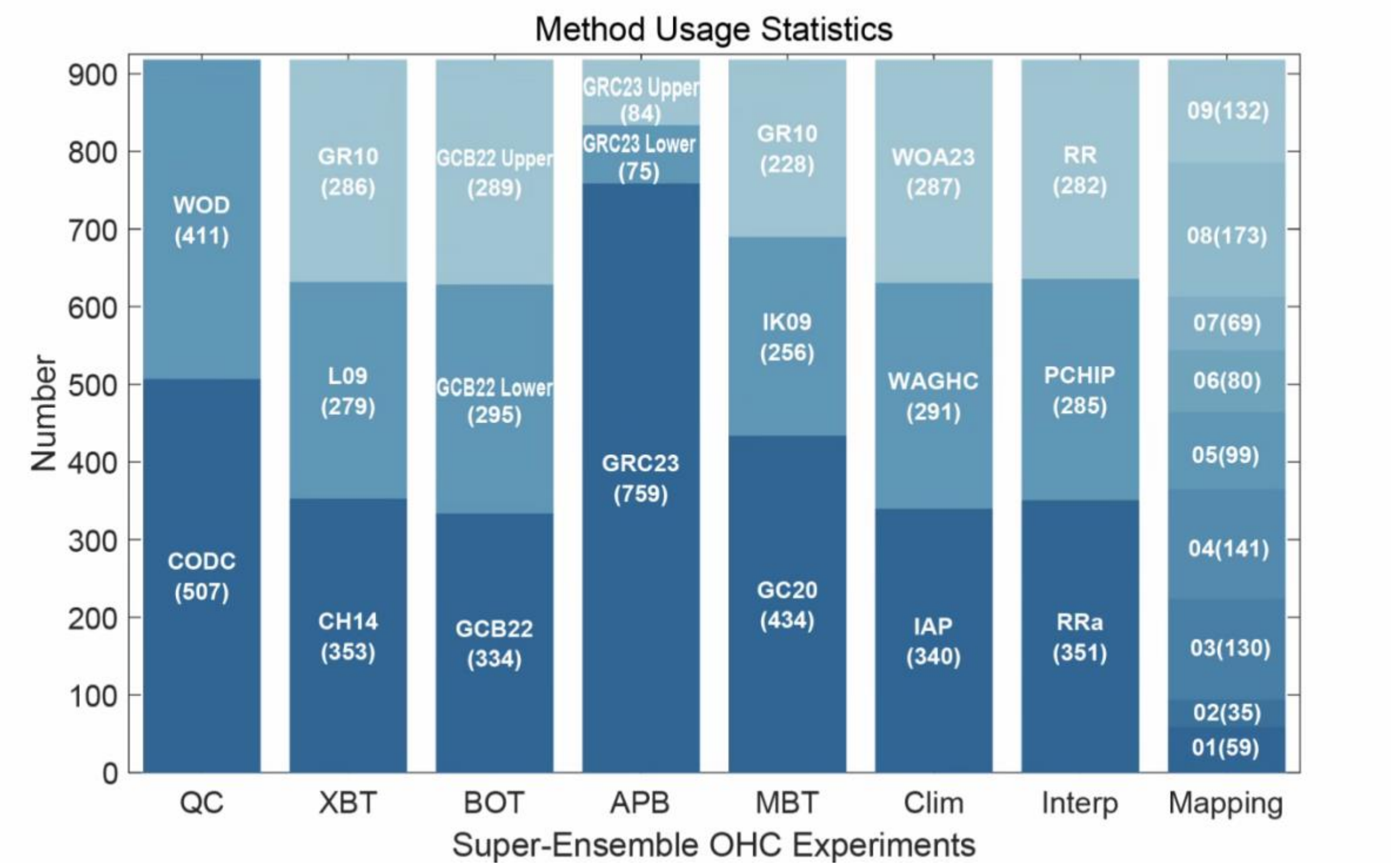

Supplementary Fig. 2. **Statistics on the use of each technique in large-ensemble.** A total of 908 datasets are included.

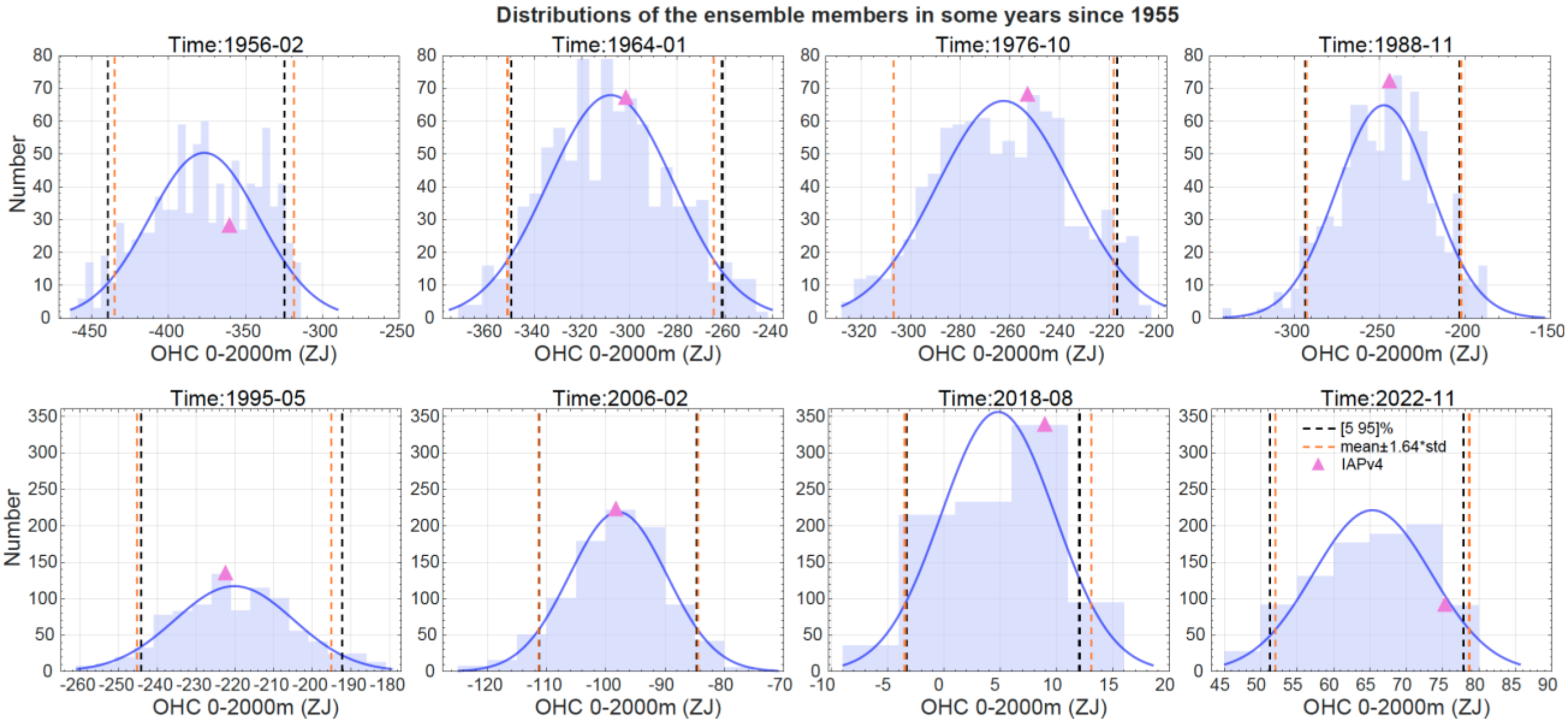

Supplementary Fig. 3. **Distributions of the ensemble members in some years since 1955**. The blue curves denote normal distribution fits of the ensemble members. The black dashed lines denote the [5%, 95%] range, and the orange dashed lines represent the mean ± 1.64 standard deviations. The magenta triangles indicate the IAPv4 estimates.

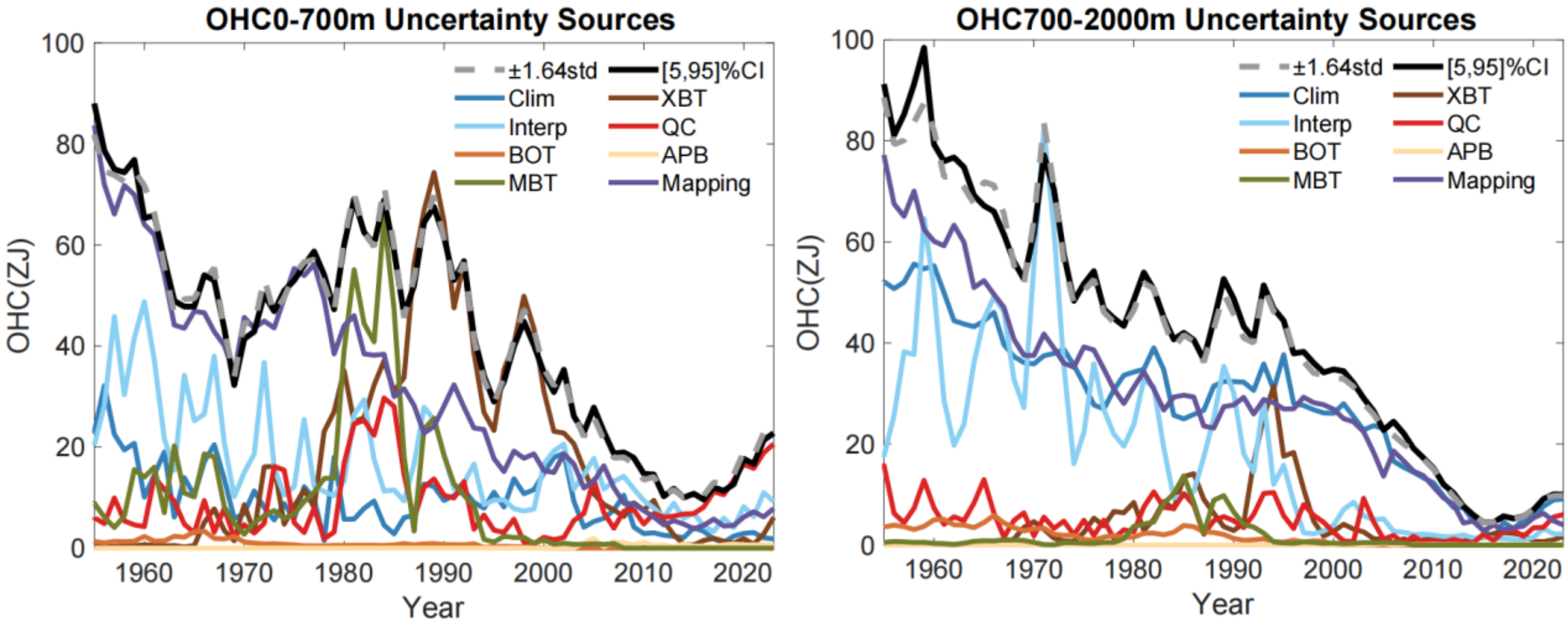

Supplementary Fig. 4. **Same as Fig. 2b but for OHC (Ocean Heat Content - OHC) 0-700m (left) and OHC700-2000m (right)**.

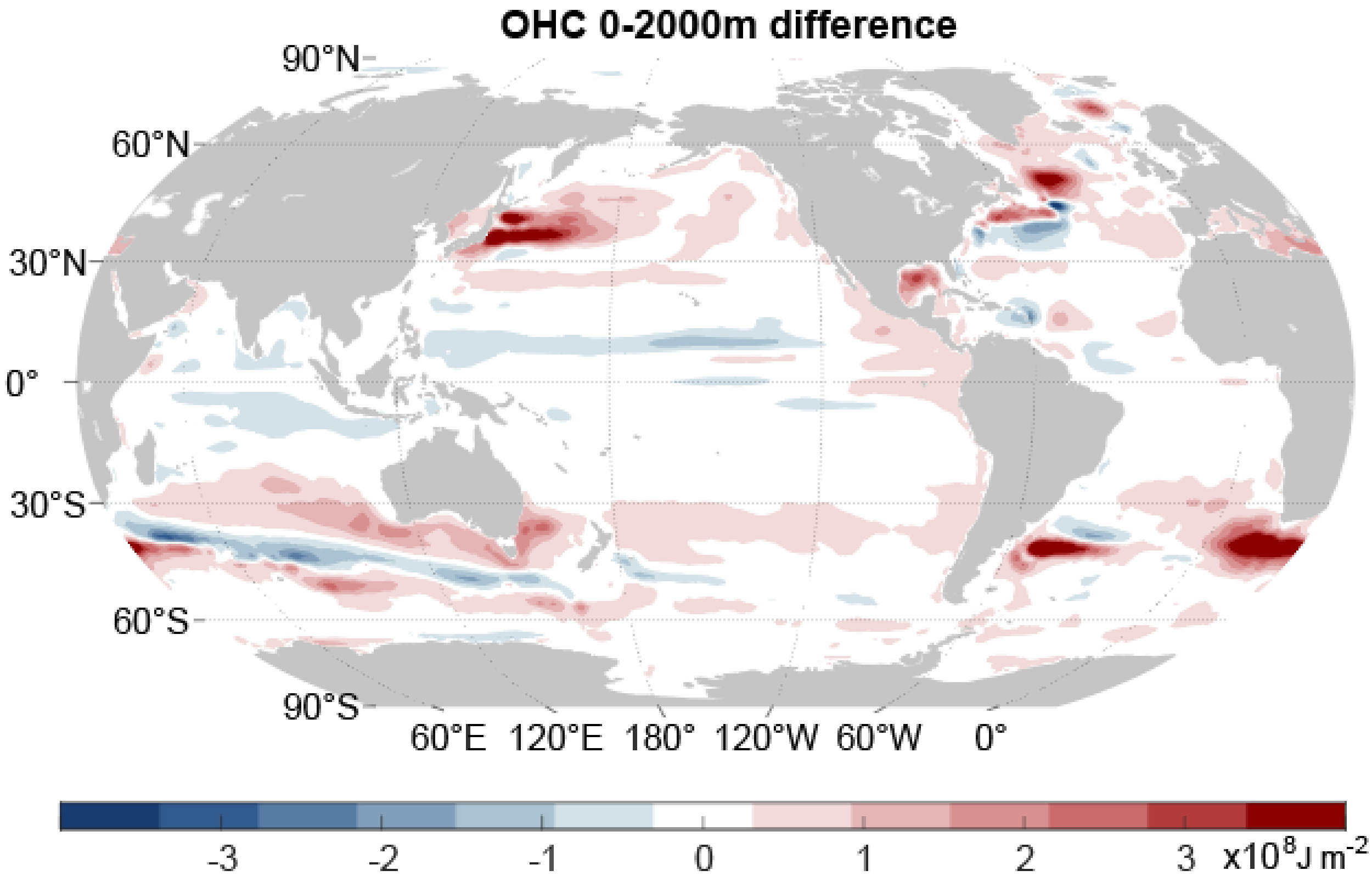

Supplementary Fig. 5. Spatial distribution of the difference in OHC (Ocean Heat Content - OHC) 0-2000m between the CODC-QC-based and WOD-QC-based estimates averaged over the period 2005–2023. Unit: J m^{-2}. (Chinese Academy of Sciences Ocean Data Center – CODC, World Ocean Database – WOD, Quality Control - QC)

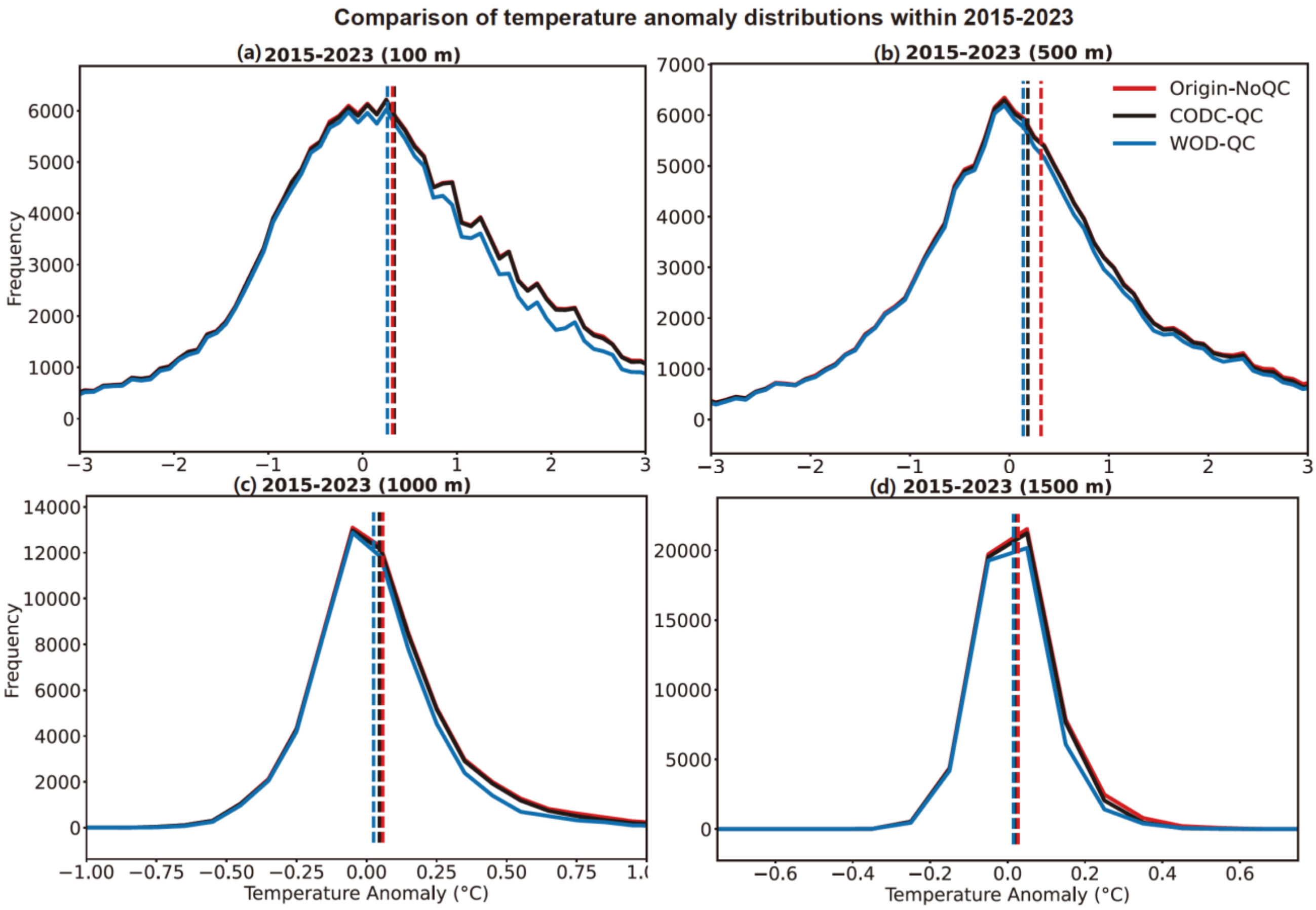

Supplementary Fig. 6. **Comparison of temperature anomaly distributions within 2015-2023 for all in situ data (red), data after WOD-QC (blue), and CODC-QC (black) in the Northwest Pacific Ocean**. These panels display the frequency distributions of temperature anomalies at depths of 100 m (a), 500 m (b), 1000 m (c), and 1500 m (d), respectively. Vertical dashed lines denote the mean of each dataset. (Chinese Academy of Sciences Ocean Data Center - CODC, World Ocean Database - WOD, Quality Control - QC)

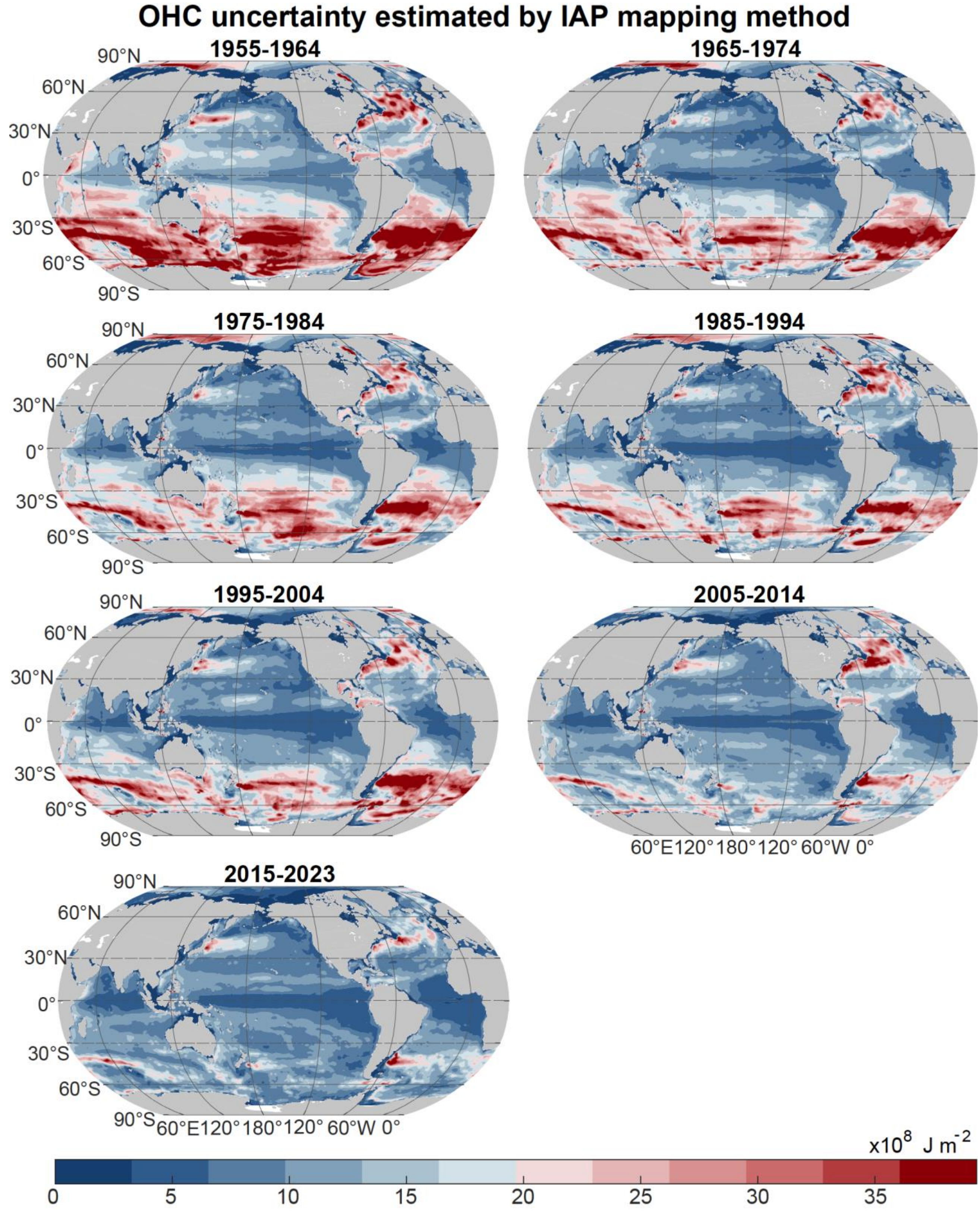

Supplementary Fig. 7. **10-year mean mapping and instrumental uncertainties (twice 1.64 times standard deviation) of OHC (Ocean Heat Content - OHC) 0–2000 m estimated from the IAP (Institute of Atmospheric Physics - IAP) mapping method. Units: J m^{-2}.**

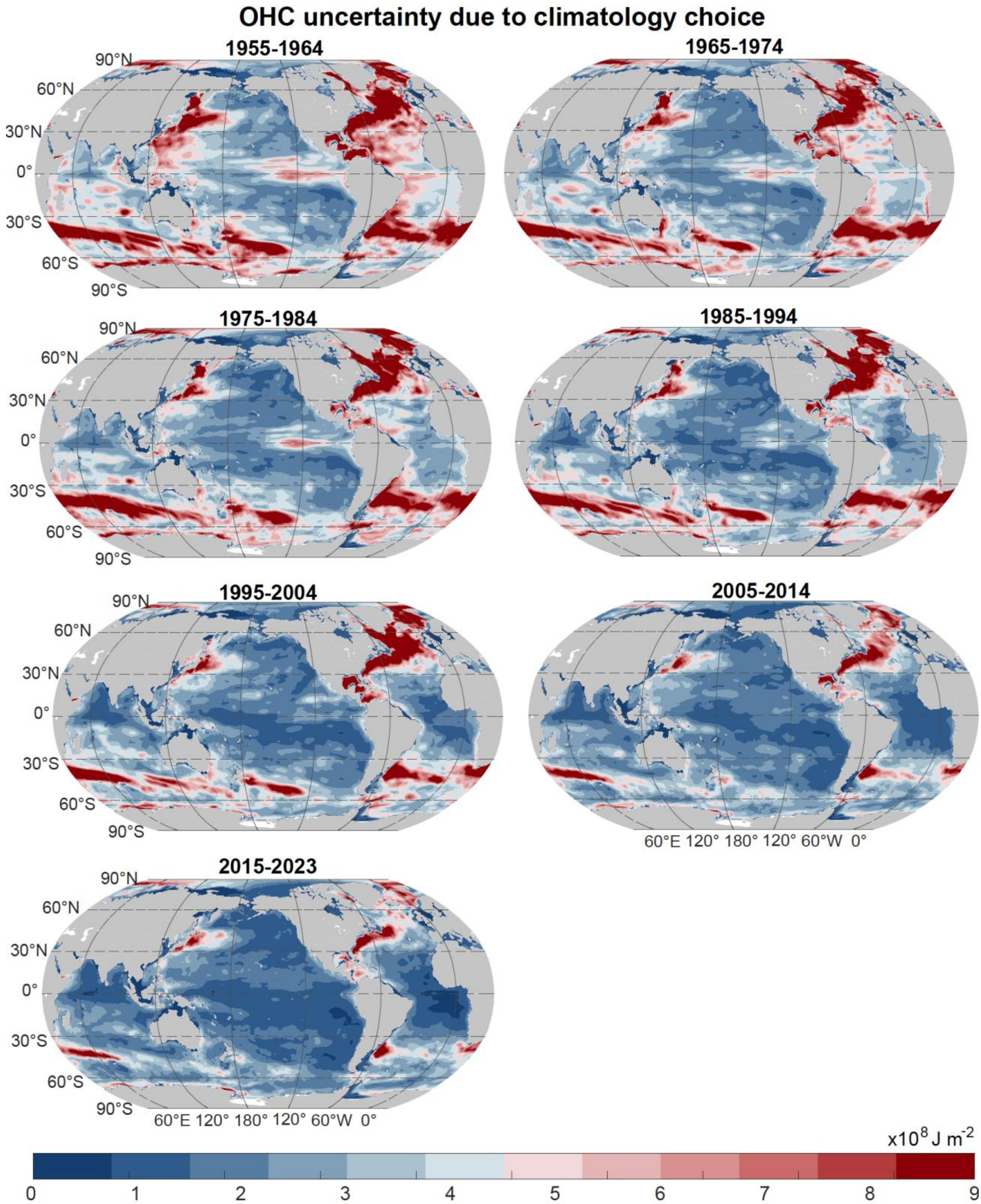

Supplementary Fig. 8. **Same as Supplementary Fig. 7 but for OHC (Ocean Heat Content - OHC) 0-2000m uncertainty caused by climatology choice.**

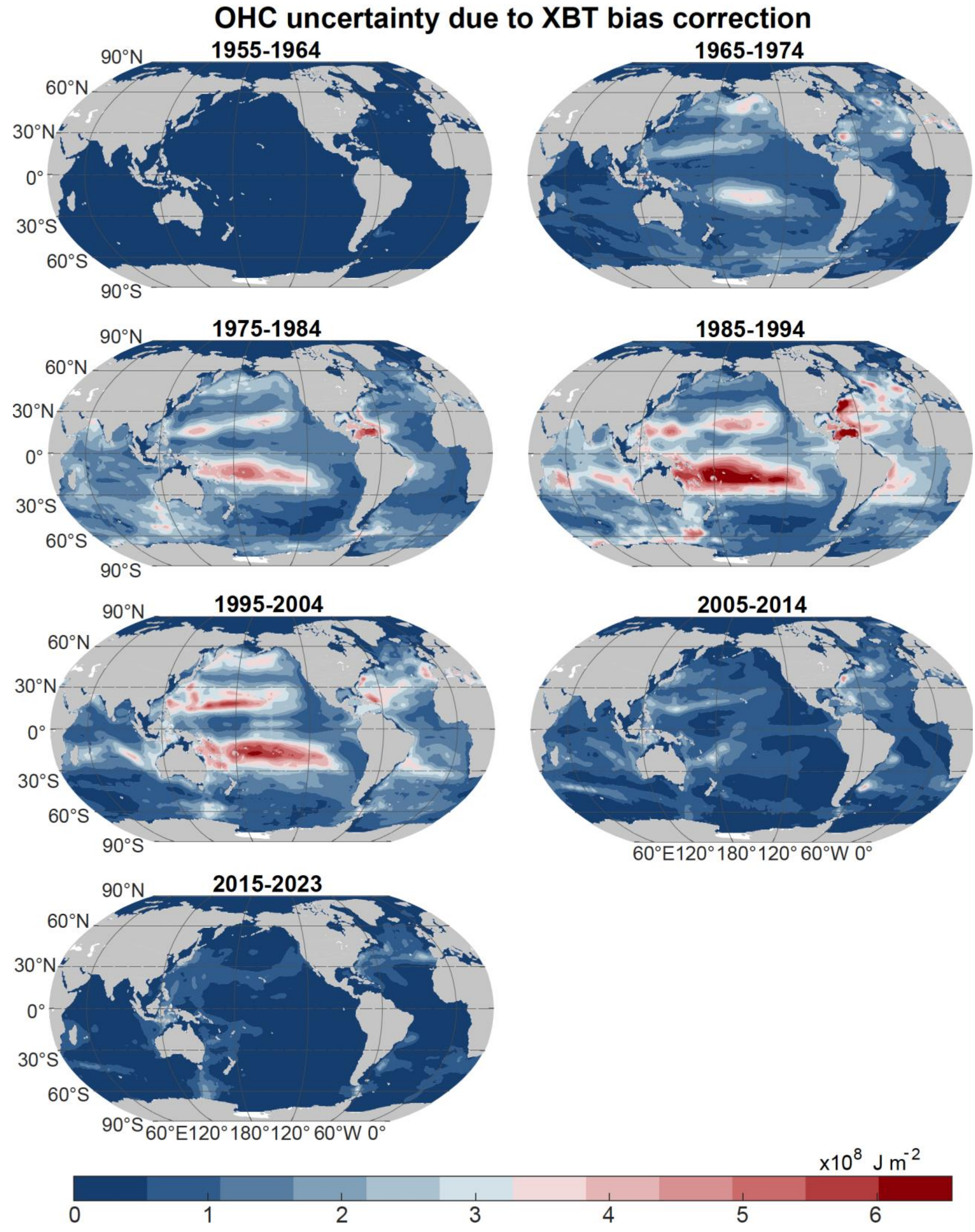

Supplementary Fig. 9. Same as Supplementary Fig. 7 but for OHC (Ocean Heat Content - OHC) 0-2000m uncertainty caused by XBT (eXpendable Bathythermographs - XBT) bias correction.

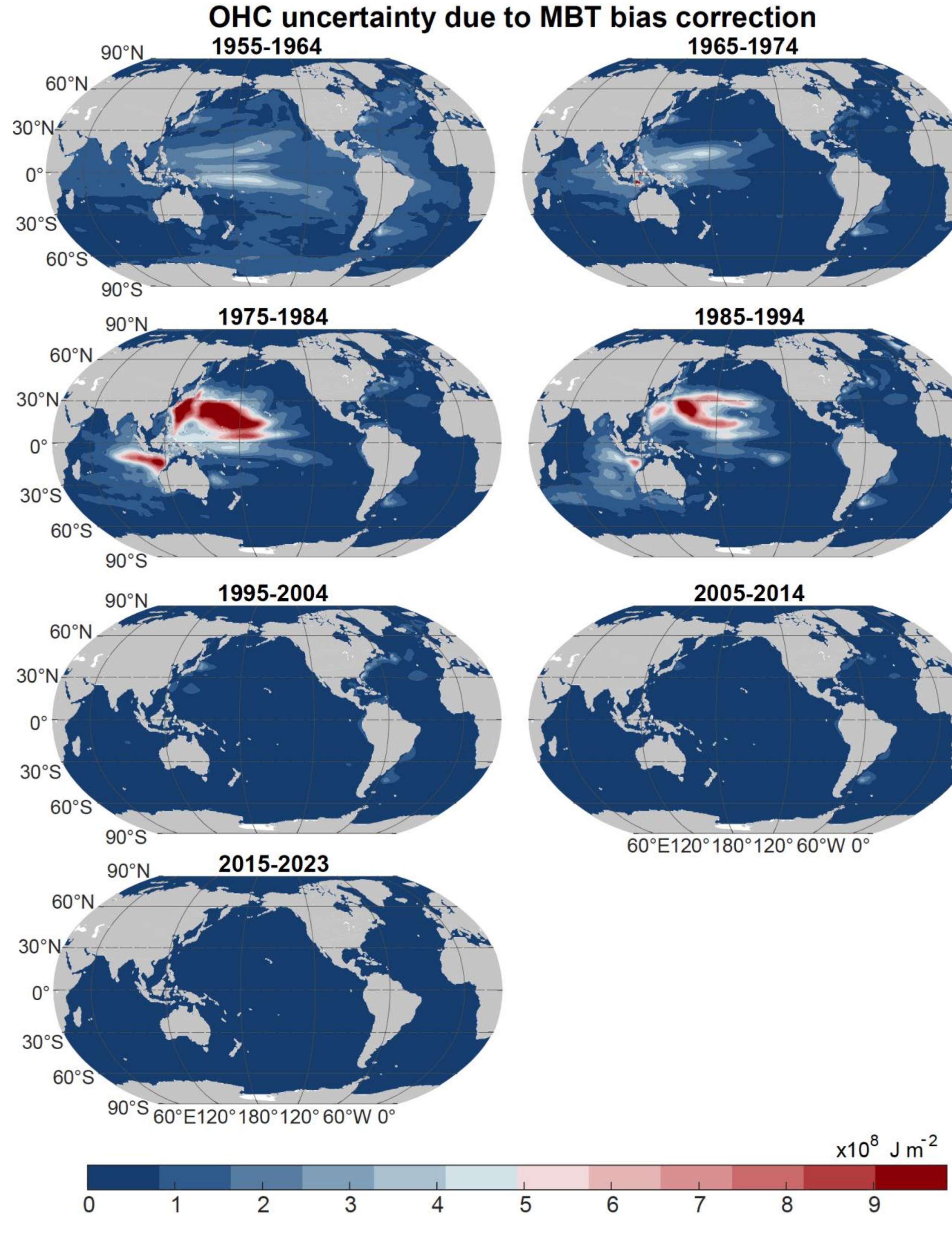

Supplementary Fig. 10. Same as Supplementary Fig. 7 but for OHC (Ocean Heat Content - OHC) 0-2000m uncertainty caused by MBT (Mechanical Bathythermographs - MBT) bias correction.

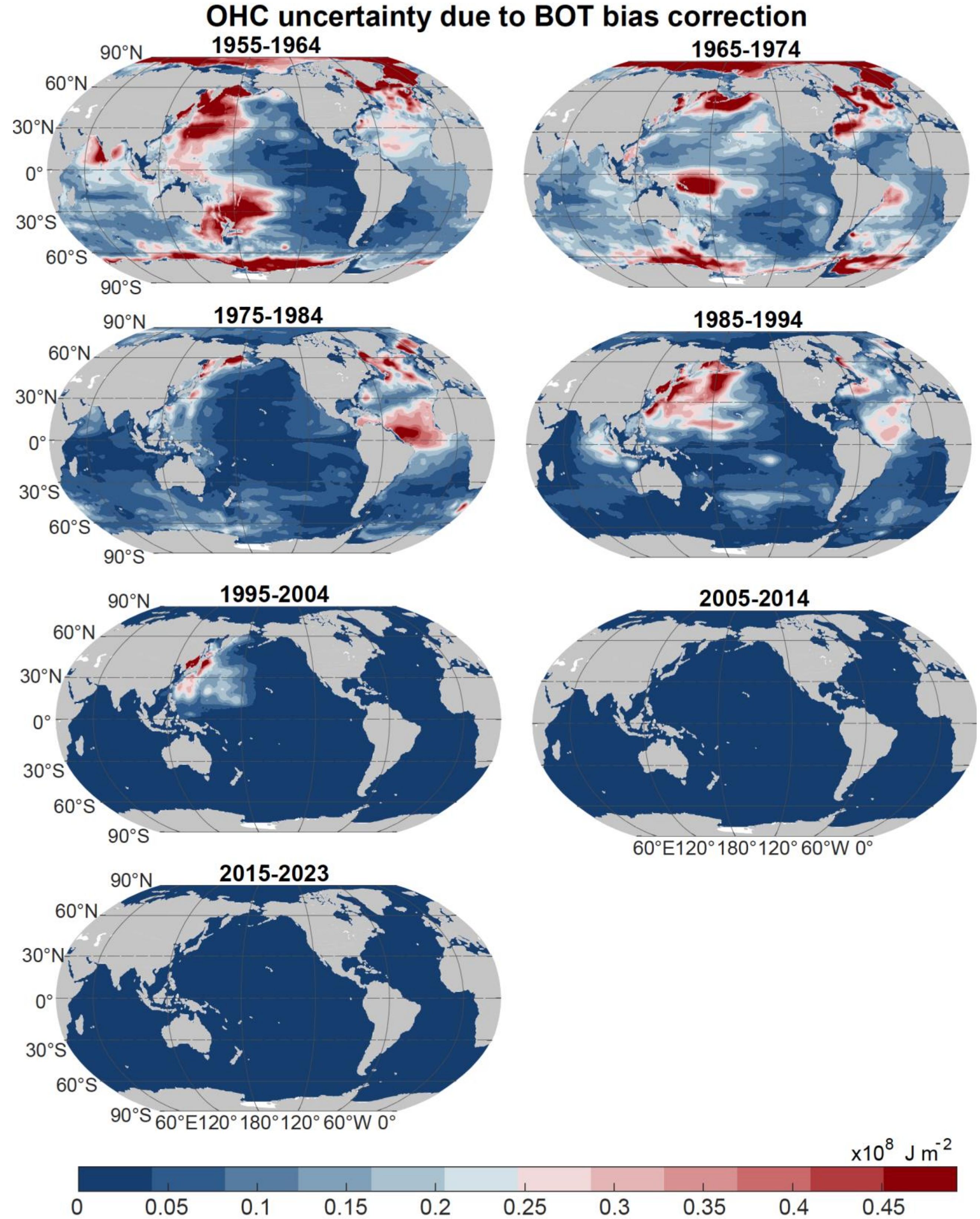

Supplementary Fig. 11. **Same as Supplementary Fig. 7 but for OHC (Ocean Heat Content - OHC) 0-2000m uncertainty caused by BOT (Bottle - BOT) bias correction**.

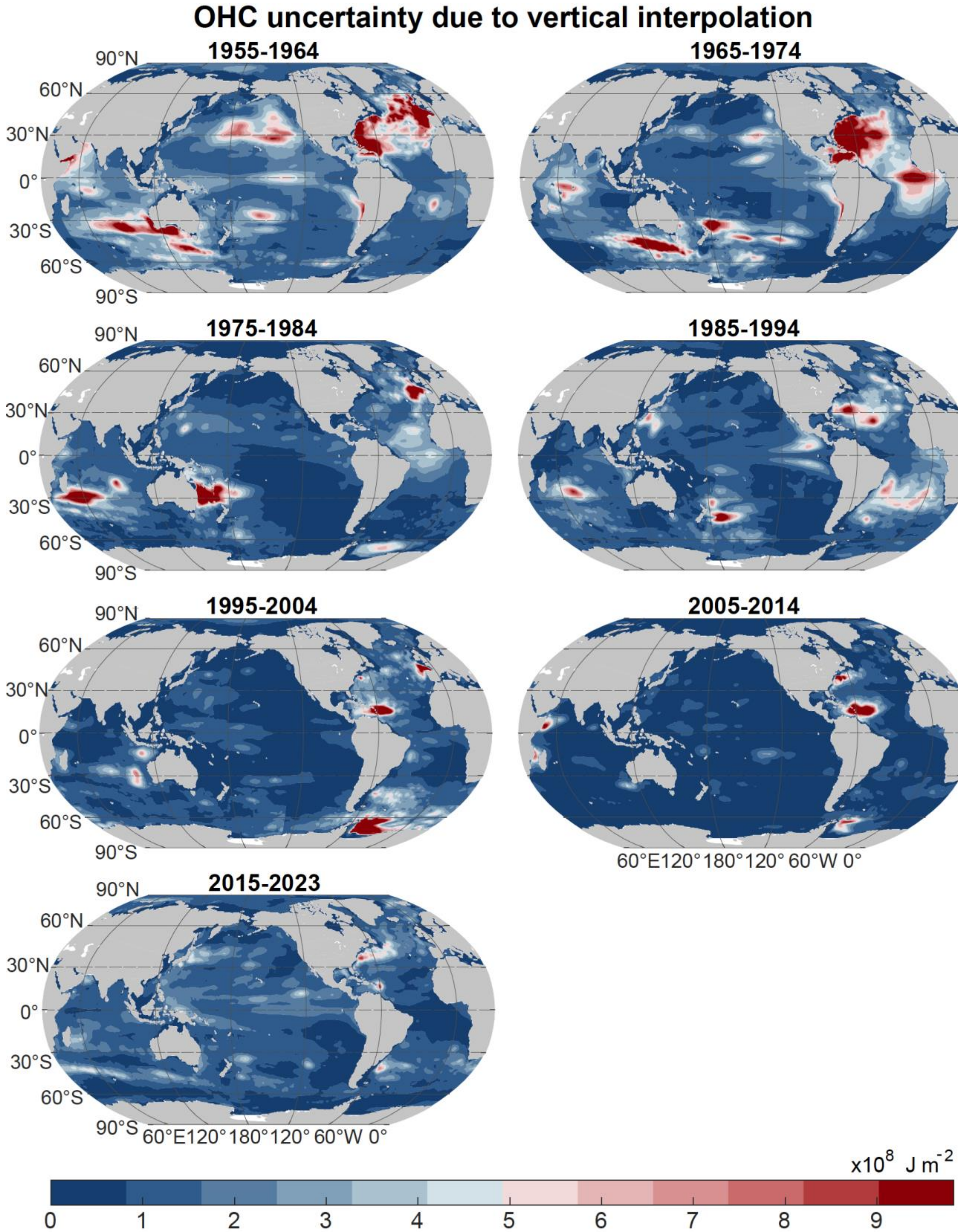

Supplementary Fig. 12. Same as Supplementary Fig. 7 but for OHC (Ocean Heat Content - OHC) 0-2000m uncertainty caused by vertical interpolation.

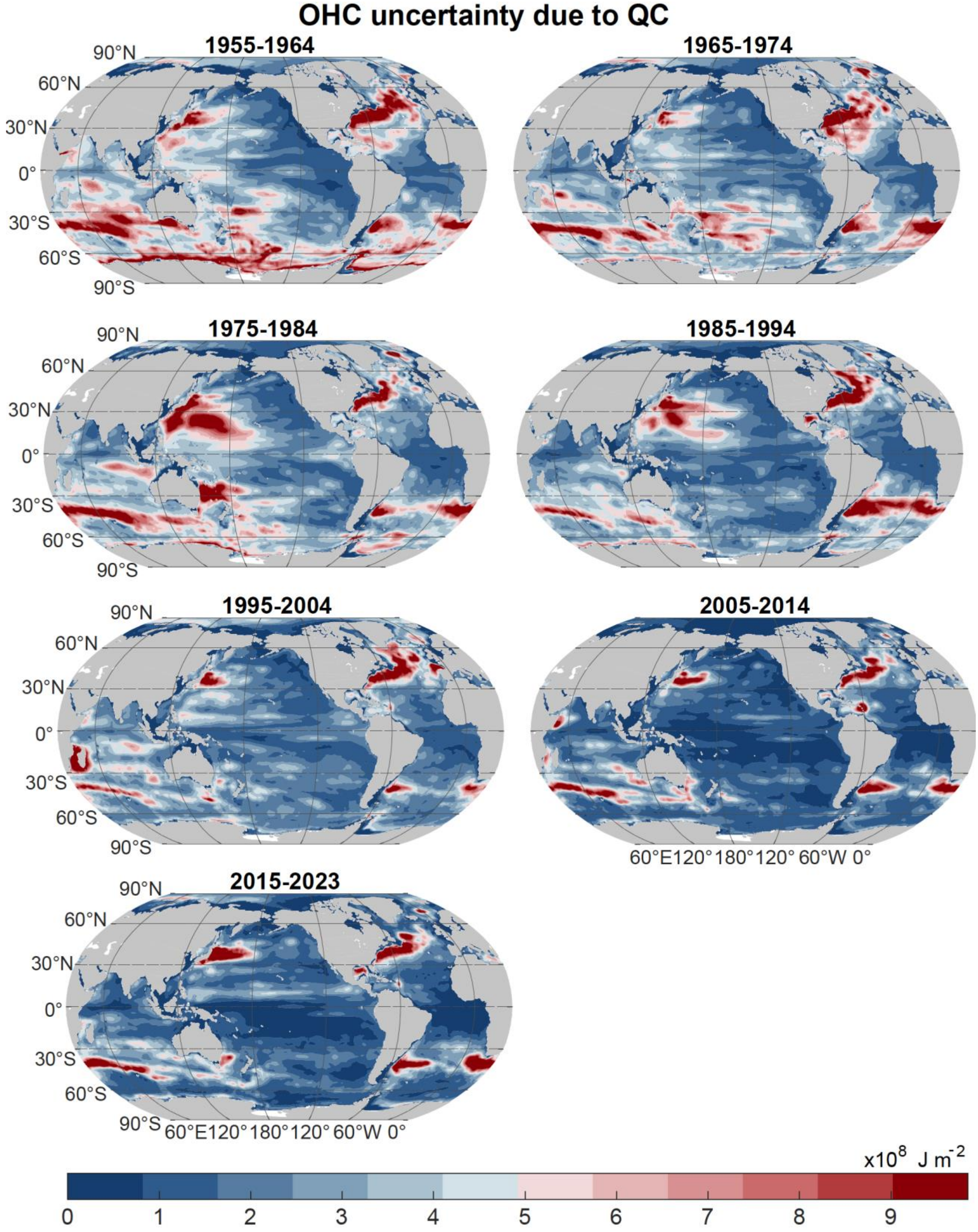

Supplementary Fig. 13. **Same as Supplementary Fig. 7 but for OHC (Ocean Heat Content - OHC) 0-2000m uncertainty caused by QC (Quality Control - QC)**.

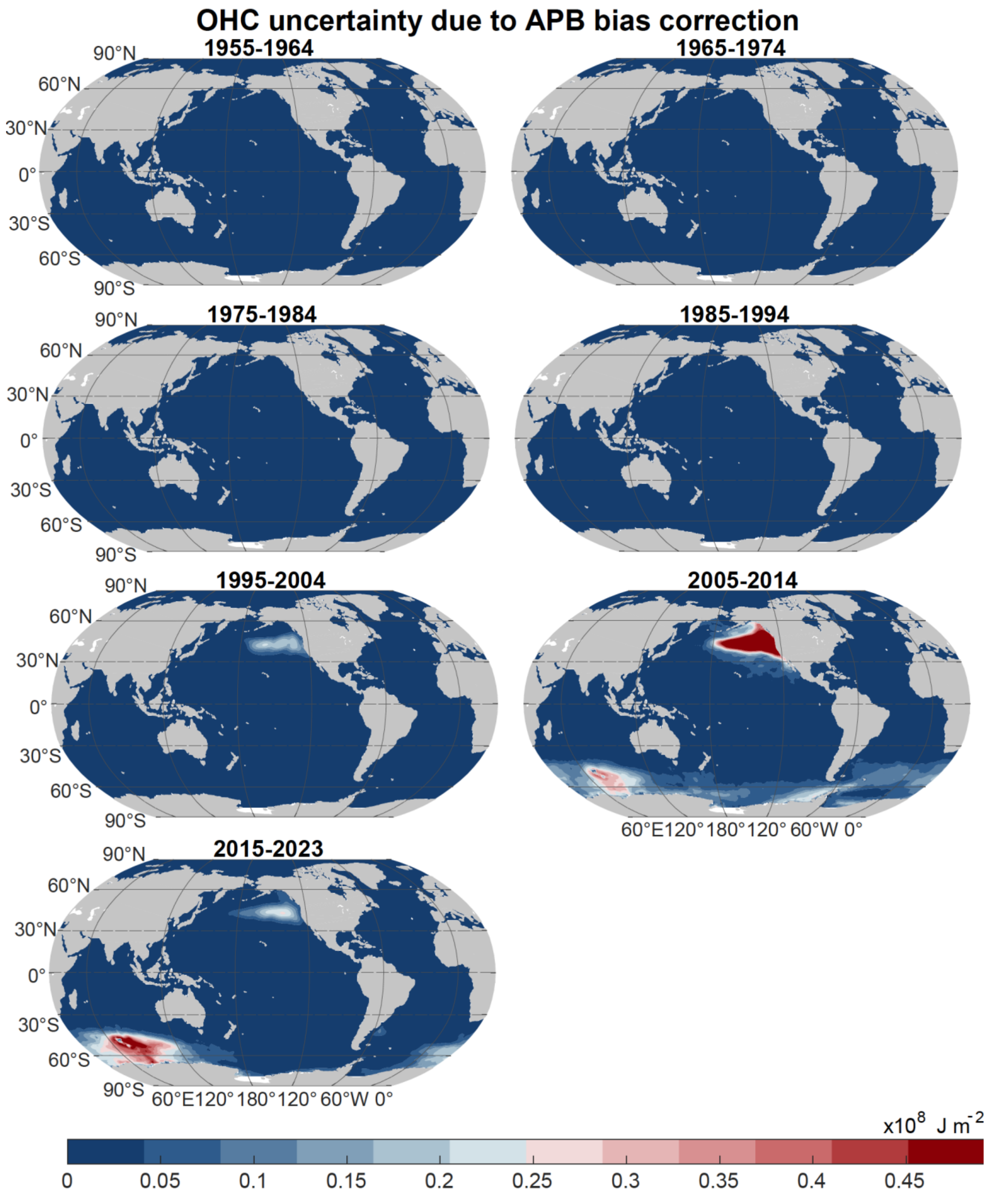

Supplementary Fig. 14. Same as Supplementary Fig. 7 but for OHC (Ocean Heat Content - OHC) 0-2000m uncertainty caused by APB (Autonomous Pinniped Bathythermographs - APB) bias correction.

Supplementary References

1 Cheng, L. & Zhu, J. Benefits of CMIP5 multimodel ensemble in reconstructing historical ocean subsurface temperature variations. *J. Clim.* **29**, 5393–5416 (2016).

2 Ablain, M. *et al.* Uncertainty in satellite estimates of global mean sea-level changes, trend and acceleration. *Earth Syst. Sci. Data* **11**, 1189–1202 (2019).
