# Supplementary material for: Six-fold reduction in ocean heat content estimate uncertainty since 1960
Source: Nat Commun. 2026 Aug 29;17:10317. doi: 10.1038/s41467-026-76436-0 (PMC13620135; doi:10.1038/s41467-026-76436-0)
Supplement: Supplementary file 2 [file 41467_2026_76436_MOESM2_ESM.pdf]

nature portfolio

Peer Review File

Six-fold reduction in ocean heat content estimate uncertainty since 1960

Corresponding Author: Professor Lijing Cheng

Parts of this Peer Review File have been redacted as indicated to remove third-party material.

This file contains all reviewer reports in order by version, followed by all author rebuttals in order by version.

Version 0:

Reviewer comments:

Reviewer #1

(Remarks to the Author)
This paper quantifies the ocean-heat-content uncertainties comprising eight contributing factors using a straightforward large-ensemble method, thereby providing a comprehensive overview of previous assessments of ocean heat content.

Although the results shown here are of limited scientific interest, the establishment of such an evaluation method is of paramount importance for the advancement of ocean science. Firstly, the variable of “ocean heat content” itself is vital for better understandings of earth system; by reducing the error bars, it is possible to capture sea-level fluctuations and the progression of global warming more accurately. Secondly, this approach provides a guideline for future global ocean observation system (GOOS). To sustain global ocean observations with limited resources, it is necessary to advance the concept of an optimal observation strategy; in this sense, this article, which addresses the dependence of the accuracy of ocean-heat-content estimation on 3-dimensional spatial resolution of data (including the identification of potential impact of eddies) and on temporal resolution of monitoring, is highly helpful.
Given the objective of this paper, the arbitrariness in the choice of the “three most up-to-date alternative techniques” for each uncertainty factor (ensemble configuration) can be considered acceptable.
Furthermore, the active discussion of the relationship between ocean heat content and Earth’s energy imbalance is important for promoting future climate research.

Major Comments:
Whilst I can find no logical inconsistencies in the structure or content, I do have one concern regarding the error covariance, which is a key variable in this paper.
The error covariance is defined by Eq. (1) as the total variance minus the sum of the individual variances. When considering the inherently non-linear relationships between the various components, is this definition justified? I’m not sure whether it’s self-evident or not. A clear explanation should be added on this point.
Provided this point is clarified, I would recommend the publication of this paper in Nature Communications.

Minor comments:
1) Line 50: “Ocean warming reduces the solubility of oxygen in the sea water, and stronger ocean stratification”
Ocean warming and stronger ocean stratification are not identical. Although I can understand if it is “surface” ocean warming, this paper does not mention changes in stratification very much. The text needs to be organised logically.

2) Line 71: “eXpendable bathythermographs (XBT)”
eXpendable BathyThermographs (XBT)

3) Line 94: “different type of errors are correlated”
different type of errors is correlated

4) Fig2c:
There appears to be a clear decadal-scale fluctuation in the temporal variation of the “interpolation” (sky blue curve; which consequently affects both the total variance and the covariance). Could you explain this? It seems to be due to a simple

cause. This effect may possibly have appeared in Figure 3(c) and (d).

5) Line 382: “Again, the sum of errors is two times larger than the total trend uncertainty, implying strong error covariance impacts on trend estimate (Fig. 6a).”
See my major comment. Is it justified?

6) L432: “(Fig. 6d)”
(Fig. 6c) ?

Reviewer #2

(Remarks to the Author)
What are the noteworthy results?

This manuscript presents several notable findings, including:

(1) A sixfold reduction in uncertainty regarding ocean heat content (OHC) since the 1950s, estimated using an ensemble method comprising 908 members;

(2) A reliable long-term increase in OHC of 499 ZJ for the first 2,000 metres since 1955, with non-overlapping uncertainty spanning multiple decades that statistically confirms the acceleration of ocean warming; and (3) A shift in the dominant sources of error over the years. While sampling was initially the main cause of uncertainty, quality control has emerged as the primary cause of uncertainty in the last decade. This is an unexpected and significant finding, attributed to the incorrect handling of the large positive temperature anomaly in eddy-rich regions by classical WOD climatology quality control.

3) A shift in the dominant sources of error over time. While it was initially mainly related to sampling, quality control has emerged as the primary cause of uncertainty in the last decade. This is an unexpected and significant finding, attributed to the incorrect handling of the large positive temperature anomaly in eddy-rich regions by classical WOD climatology quality control methods.
The structure of covariance errors was also examined, revealing that individual sources of error are not independent and that their sum far exceeds the total uncertainty. If covariance is ignored, the total uncertainty is ultimately overestimated. Furthermore, the dominance of error covariance over the past decade is highly informative.

5) The trend of 10.9 ZJ/yr for the years 2005–2023 and the acceleration of 0.34 W/m^2/decade are both statistically significant across the ensemble members.

6) The regional uncertainty maps for the last six decades provide important information and guidance for the global ocean monitoring community.

Will the work be of significance to the field and related fields? How does it compare to the established literature? If the work is not original, please provide relevant references.

This work is significant and original, providing the most comprehensive assessment of uncertainty in ocean heat content (OHC) to date. It surpasses previous studies, such as those by Layman et al. (2010, Nature), which provided an initial estimate and covered four sources for the period from 1993 to 2008 in the first 700 metres. Boyer et al. (2016) provide three sources without estimating total uncertainty and ignore covariance, covering the period from 1970 to 2008. Sevita et al. (2022) also identify three sources of uncertainty at a local rather than global scale for the period 1970–2008, while again ignoring covariance. This study acknowledges the limitations of the above works and examines them systematically. The finding that quality control has surpassed sampling as the primary source of errors since 2010 is novel and has not been reported elsewhere to date.

Does the work support the conclusions and claims, or is additional evidence needed?

The data largely support the conclusions, with some reservations: The 908 members of the method are well designed and converge, which supports the reliability of the method for estimating uncertainty. The nearly Gaussian distribution of the members confirms the interpretation of the quantiles. The agreement between the two independent estimation methods for the uncertainty trend is a strong indication of reliability. However, the comparison with CERES-EEI, which has a correlation coefficient of 0.6 for dOHC/dt that improves to 0.71 when the atmospheric component is included, is satisfactory but insufficient. Furthermore, interannual discrepancies have been identified but remain unresolved. The claim that WOD quality control introduces a systematic bias in OHC in eddy-rich regions is significant. It would be important to conduct a quantitative assessment using independent observations in specific regions. The assertion that CODC quality control represents the 'best available knowledge' when used as the sole quality control option must be verified independently. Only IAP mapping was used due to the unavailability of the code for the other methods. Although the En-OI approach is well documented and yields consistent results, this remains a significant limitation for uncertainty mapping.

Are there any flaws in the data analysis, interpretation and conclusions? Do these prohibit publication or require revision?

While there are no major omissions that call the results into question, some issues warrant further review:

The authors acknowledge the limitation of using only two quality control systems, which suggests that uncertainty from quality control is underestimated or not adequately specified. The two systems are not independent (the CODC was developed by the same group), so potential bias must be discussed. Secondly, using only the IAP's EnOI for mapping uncertainty, even with cross-validation of resampling, raises a concern as the IAP's mapping is also the central estimate. The authors should provide more extensive commentary on whether this leads to an underestimation of uncertainty mapping.The discussion correctly notes that the method cannot capture the 'unknown unknowns', but this is only mentioned briefly. Given that this paper emphasises accuracy (e.g. ~10 ZJ, the best estimate since 2015), this limitation deserves greater emphasis and a lower bound should be provided wherever possible. In Figure 2b, the observed increase is attributed exclusively to quality control. However, a thorough analysis of its origin might provide a clearer picture. For example, if one of the two quality control systems were removed, would the effect on the uncertainty trend decrease? In Equation 1 for the covariance of the error, no reference is made to the statistical methodology. A brief explanation would help to make the method easier to reproduce. Points 1–4 of the discussion are useful and could be expanded upon.

Is the methodology sound? Does the work meet the expected standards in your field?

The methodology is robust and sophisticated. Its main strengths are as follows: The large ensemble approach utilises three leading techniques for error sources and is well-documented and computationally feasible. This avoids the artificial inflation of error caused by outdated methods. The separation of uncertainty estimates for the annual mean OHC, the trend and the dOHC/dt is methodologically significant and has been correctly implemented. The variance–covariance matrix framework (Equation 2) for time-correlated errors is rigorous and appropriate, and the use of LOWESS for trend estimation with matrix method cross-validation is considered best practice in this field. The decision to limit the number of ensemble members to ~900 (rather than the full 4,374) is well justified by the demonstrated convergence. One comment on the methodology is that selecting three methods per error source implies that three samples are enough to adequately represent the distribution of each source. While this may be justified for some methods, such as vertical regression and climatology, based on the available literature, for others, such as XBT correction, a more rigorous selection process is required. However, the authors do address this in the 'Methods' chapter.

Is there enough detail provided in the methods for the work to be reproduced?

It is partially satisfactory, but improvements are needed. The raw in-situ data (WOD/CODC) and CERES products are publicly available and cited. The dataset is also available on the IAP FTP server, which is commendable. However, the main shortcoming is the unavailability of the code. The mapping code (IAP EnOI) is not documented sufficiently to allow reproduction. The dataset construction process, which incorporates eight error sources across 908 members, should have an associated code repository. While the article states that the codes for alternative mapping approaches 'are not publicly available', the availability of the authors' own code is not explicitly mentioned. Certain parameters (e.g. the set of 30 CMIP5 models used in EnOI and the climatology construction details for each of the three methods) are mentioned, but not described with enough detail to allow independent reproduction. For full reproducibility, a supplementary methods section with greater technical depth would be necessary.

I recommend accepting the manuscript after making the following minor revisions.

1) A statement must be included regarding the availability of the code.
2) The fact that both systems originate from the IAP/CAS must be explicitly mentioned in relation to the independence of quality control.
3) The restriction to using only one method for matching should be clarified more clearly in the abstract and conclusions.
4) The CODC-QC claim regarding 'best available knowledge' should be supported by independent validation.
5) Standardise abbreviations, expand the statistical basis of equation 1 and qualify the phrase 'best estimate' for the 10 ZJs to reflect remaining structural uncertainties.

Version 1:

Reviewer comments:

Reviewer #1

(Remarks to the Author)
I am satisfied with the author's response including their handling of my major comment.
I acknowledge that the revisions are both logically and scientifically sound, and I recommend acceptance of the paper.

(Remarks on code availability)

Reviewer #2

(Remarks to the Author)
The authors have addressed all of my comments adequately. I am completely satisfied with the clarifications, changes and additions made to the revised manuscript. For this reason, I recommend that it be published without any further changes.

(Remarks on code availability)
Although I am not a Python expert, I found the code understandable and usable, and it can be run by an average user. The ReadMe file is not very long, but it provides basic information about the script.

Open Access This Peer Review File is licensed under a Creative Commons Attribution 4.0 International License, which permits use, sharing, adaptation, distribution and reproduction in any medium or format, as long as you give appropriate credit to the original author(s) and the source, provide a link to the Creative Commons license, and indicate if changes were made.
In cases where reviewers are anonymous, credit should be given to 'Anonymous Referee' and the source.
The images or other third party material in this Peer Review File are included in the article’s Creative Commons license, unless indicated otherwise in a credit line to the material. If material is not included in the article’s Creative Commons license and your intended use is not permitted by statutory regulation or exceeds the permitted use, you will need to obtain permission directly from the copyright holder.
To view a copy of this license, visit https://creativecommons.org/licenses/by/4.0/

We'd like to thank three reviewers for the constructive comments. We have revised the manuscript accordingly. Below we provide a point-by-point response to each reviewer's comments. Reviewer comments are shown in **black**, our responses in **blue**, and changes to the manuscript are indicated in **orange**.

REVIEWER COMMENTS

Reviewer #1 (Remarks to the Author):

This paper quantifies the ocean-heat-content uncertainties comprising eight contributing factors using a straightforward large-ensemble method, thereby providing a comprehensive overview of previous assessments of ocean heat content.

Although the results shown here are of limited scientific interest, the establishment of such an evaluation method is of paramount importance for the advancement of ocean science. Firstly, the variable of "ocean heat content" itself is vital for better understandings of earth system; by reducing the error bars, it is possible to capture sea-level fluctuations and the progression of global warming more accurately. Secondly, this approach provides a guideline for future global ocean observation system (GOOS). To sustain global ocean observations with limited resources, it is necessary to advance the concept of an optimal observation strategy; in this sense, this article, which addresses the dependence of the accuracy of ocean-heat-content estimation on 3-dimensional spatial resolution of data (including the identification of potential impact of eddies) and on temporal resolution of monitoring, is highly helpful.
Given the objective of this paper, the arbitrariness in the choice of the "three most up-to-date alternative techniques" for each uncertainty factor (ensemble configuration) can be considered acceptable.
Furthermore, the active discussion of the relationship between ocean heat content and Earth's energy imbalance is important for promoting future climate research.
Re: Thanks for the evaluation of this study.

Major Comments:
Whilst I can find no logical inconsistencies in the structure or content, I do have one concern regarding the error covariance, which is a key variable in this paper.
The error covariance is defined by Eq. (1) as the total variance minus the sum of the individual variances. When considering the inherently non-linear relationships between the various components, is this definition justified? I'm not sure whether it's self-evident or not. A clear explanation should be added on this point.
Provided this point is clarified, I would recommend the publication of this paper in Nature Communications.
Re: Thanks for pointing out this issue. Strictly, covariance in statistics defines the linear relationship between two variables (linear association), thus our "covariance" after Eq.(1), that is, the total variance minus the sum of individual variances, indeed includes

measures of non-linear relationships between two variables. However, there is no proper wording used to define the complex relationships to our knowledge, and covariance is easier to understand, thus we decided to keep the wording “covariance” in the paper but explicitly explain after Eq.(1) that our “covariance” includes both linear and nonlinear relationships between variables.

Minor comments:

1) Line 50: “Ocean warming reduces the solubility of oxygen in the sea water, and stronger ocean stratification”
Ocean warming and stronger ocean stratification are not identical. Although I can understand if it is “surface” ocean warming, this paper does not mention changes in stratification very much. The text needs to be organised logically.
Re: this sentence is simplified to “Ocean warming reduces the solubility of oxygen in the seawater and contributes to the ocean deoxygenation, threatening ocean life and marine fisheries.”

2) Line 71: “eXpendable bathythermographs (XBT)”
eXpendable BathyThermographs (XBT)
Re: Modified.

3) Line 94: “different type of errors are correlated”
different type of errors is correlated
Re: Modified to “types of errors are correlated”.

4) Fig2c: There appears to be a clear decadal-scale fluctuation in the temporal variation of the “interpolation” (sky blue curve; which consequently affects both the total variance and the covariance). Could you explain this? It seems to be due to a simple cause. This effect may possibly have appeared in Figure 3(c) and (d).
Re: Thanks, and we have offered some explanations in the revised manuscript. As shown in the figure of the global mean vertical resolution (Fig. X1), in some years, such as ~1960 and ~1970 and ~1980, there are large amounts of low-resolution data, which contribute to increased uncertainty due to vertical interpolation. Thus, the change in data vertical resolution can generally explain the uncertainty variability. However, the impacts of vertical resolution also depend on other factors such as the spatial distribution of data; for instance, if a low-resolution profile is in a data-sparse area, the impacts will be much bigger than when it is surrounded by many high-resolution data. A brief discussion is added in the paper.

[FIGURE REDACTED]

5) Line 382: “Again, the sum of errors is two times larger than the total trend uncertainty, implying strong error covariance impacts on trend estimate (Fig. 6a).”. See my major comment. Is it justified?

Re: It is an interesting result that the sum of errors is almost twice the total trend uncertainty, indicating that different techniques strongly interact. We provide several examples to understand this in the manuscript. One of them is: even though there is a bad QC that does not remove erroneous data (QC uncertainty), the mapping methods have an effect of smoothing that can ease the QC impact. Another example is the impact of vertical resolution, as discussed in the previous reply. Thus, a smaller total trend uncertainty than the sum of individual errors is expected. What is a bit surprising is the magnitude of the difference; we believe it deserves some in-depth analysis.

6) L432: “(Fig. 6d)” (Fig. 6c) ?

Re: Corrected.

Reviewer #2 (Remarks to the Author):

What are the noteworthy results?

This manuscript presents several notable findings, including:

(1) A sixfold reduction in uncertainty regarding ocean heat content (OHC) since the 1950s, estimated using an ensemble method comprising 908 members;

(2) A reliable long-term increase in OHC of 499 ZJ for the first 2,000 metres since 1955, with non-overlapping uncertainty spanning multiple decades that statistically confirms the acceleration of ocean warming; and (3) A shift in the dominant sources of error over the years. While sampling was initially the main cause of uncertainty, quality control has emerged as the primary cause of uncertainty in the last decade. This is an unexpected and significant finding, attributed to the incorrect handling of the large positive temperature anomaly in eddy-rich regions by classical WOD climatology quality control.

3) A shift in the dominant sources of error over time. While it was initially mainly related to sampling, quality control has emerged as the primary cause of uncertainty in the last decade. This is an unexpected and significant finding, attributed to the incorrect handling of the large positive temperature anomaly in eddy-rich regions by classical WOD climatology quality control methods.

The structure of covariance errors was also examined, revealing that individual sources of error are not independent and that their sum far exceeds the total uncertainty. If covariance is ignored, the total uncertainty is ultimately overestimated. Furthermore, the dominance of error covariance over the past decade is highly informative.

5) The trend of 10.9 ZJ/yr for the years 2005–2023 and the acceleration of 0.34 W/m^2/decade are both statistically significant across the ensemble members.

6) The regional uncertainty maps for the last six decades provide important information and guidance for the global ocean monitoring community.

Re: Thanks for the thorough evaluation of our study.

Will the work be of significance to the field and related fields? How does it compare to the established literature? If the work is not original, please provide relevant references.

This work is significant and original, providing the most comprehensive assessment of uncertainty in ocean heat content (OHC) to date. It surpasses previous studies, such as those by Layman et al. (2010, Nature), which provided an initial estimate and covered four sources for the period from 1993 to 2008 in the first 700 metres. Boyer et al. (2016) provide three sources without estimating total uncertainty and ignore covariance, covering the period from 1970 to 2008. Sevita et al. (2022) also identify three sources of uncertainty at a local rather than global scale for the period 1970–2008, while again ignoring covariance. This study acknowledges the limitations of the above works and examines them systematically. The finding that quality control has surpassed sampling as the primary source of errors since 2010 is novel and has not been reported elsewhere to date.

Re: Thanks for the thorough evaluation of our study.

Does the work support the conclusions and claims, or is additional evidence needed?

The data largely support the conclusions, with some reservations: The 908 members of the method are well designed and converge, which supports the reliability of the method for estimating uncertainty. The nearly Gaussian distribution of the members confirms the interpretation of the quantiles. The agreement between the two independent estimation methods for the uncertainty trend is a strong indication of reliability. However, the comparison with CERES-EEI, which has a correlation coefficient of 0.6 for dOHC/dt that improves to 0.71 when the atmospheric component is included, is satisfactory but insufficient. Furthermore, interannual discrepancies have been identified but remain unresolved. The claim that WOD quality control introduces a systematic bias in OHC in eddy-rich regions is significant. It would be important to conduct a quantitative assessment using independent observations in specific regions. The assertion that CODC quality control represents the 'best available knowledge' when used as the sole quality control option must be verified independently. Only IAP mapping was used due to the unavailability of the code for the other methods. Although the En-OI approach is well documented and yields consistent results, this remains a significant limitation for uncertainty mapping.

Re: Thanks for the comments.

(1). For the improvement of correlation between dOHC/dt+atmospheric heat content and CERES-EEI correlation, which is also found by Mayer et al. 2025. We fully agree that “interannual discrepancies have been identified but remain unresolved”, which is an active area of research, and we explicitly state this issue in the conclusion and discussion section. But this study showed that the 8 groups of error sources could at least partly account for a substantial amount of the discrepancy (Fig. 2 and Fig. 6).

(2). We agree that it is too strong to claim that CODC-QC is the best; in our uncertainty quantification, we treated CODC-QC and WOD-QC equally, so they both form the Large-Ensemble. And we suggested in the final section that “The data quality and QC procedure should be further improved (Figs. 2,6), including a way to investigate why QC leads to recent substantial uncertainty, what the best QC practices are, and how to remove the systematic errors in ocean observations.”. We have removed the sentence of “Thus, the WOD-QC-based OHC estimates are biased and show a systematically lower rate of warming in the recent decade.” and added a figure in supplementary to support “An investigation indicates that the WOD local climatological range in its QC check, which is constructed by all historical data, mainly represents the historical ocean conditions and thus removes more positive but realistic temperature anomalies in the eddy-rich regions (such as Boundary Currents and Antarctic Circumpolar Currents regions) than CODC (Figs. S5, S6)”. That is the data distribution in a region of the Northwest Pacific Ocean before QC, after CODC-QC, and after WOD-QC. One can see that more positive data have been removed by WOD-QC. And we are now organizing a study to comprehensively evaluate the two QC systems (Fig. S6).

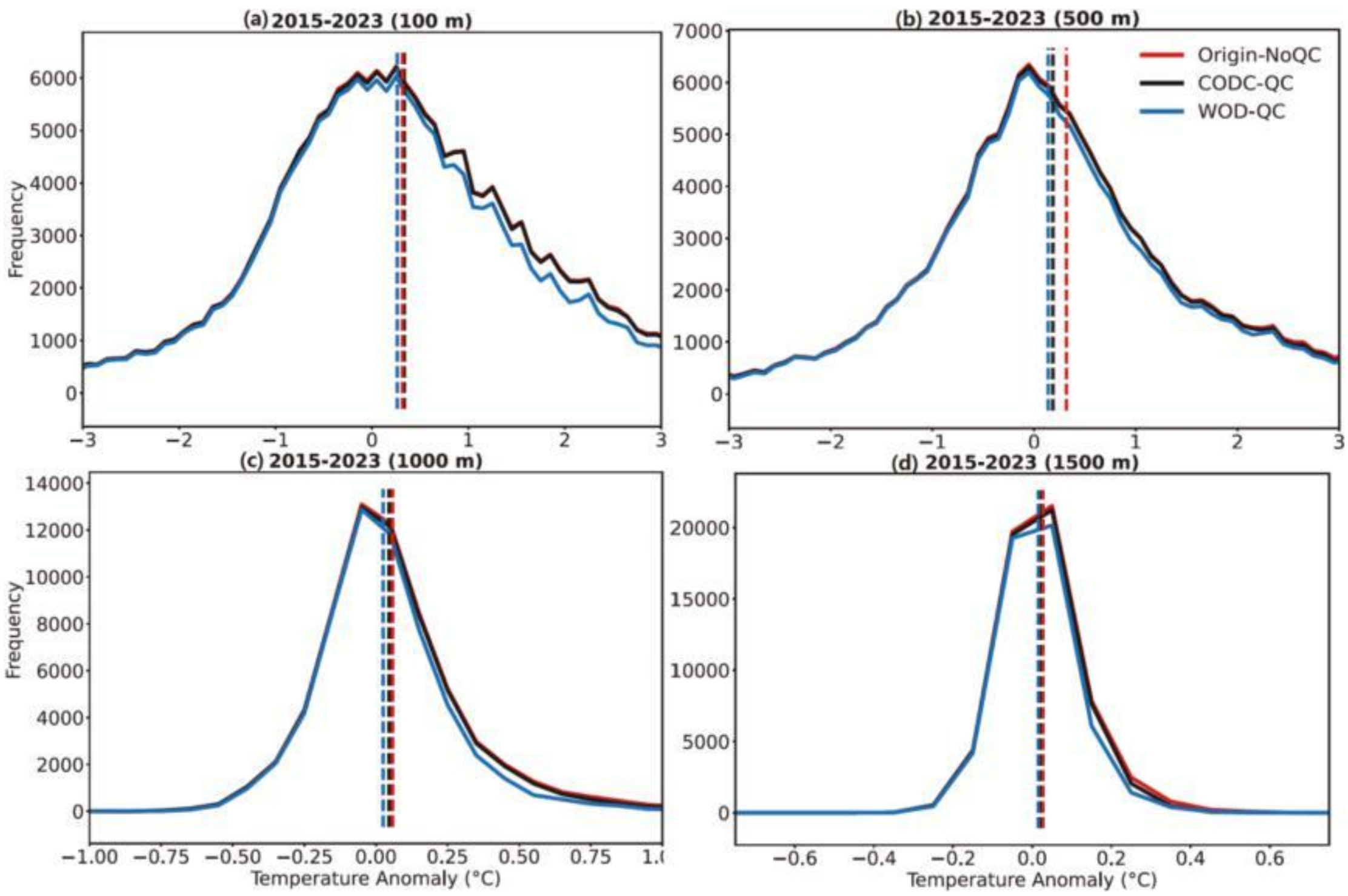

Figure S6. Comparison of temperature anomaly distributions within 2015–2023 for all in situ data (red), data after CODC-QC (black), and WOD-QC (blue) in the Northwest Pacific Ocean (28N-42N 140E -180). These panels display the frequency distributions of temperature anomalies at depths of 100 m (a), 500 m (b), 1000 m (c), and 1500 m (d), respectively. Vertical dashed lines denote the mean.

(3). Using one mapping method does not necessarily be a limitation because different mapping methods are associated with different uncertainty, for instance, Lyman and Johnson 2008 pointed out that the two global mean calculation methods: simple area integral (SI) and weighted integral (WI) perform very differently and are associated with different uncertainty (SI is heavily biased and then leads to much bigger OHC uncertainty than using WI method). Thus, the mapping uncertainty is indeed method-specific and should not be adequately captured by the spread across various mapping approaches.

As the primary goal of mapping is to fill the data gaps. The key difficulty for quantifying sampling/mapping uncertainty is that the truth in the under-sampled regions will never be known, so there must be some indirect approaches.
An additional feasible approach is the re-sampling method used in Cheng et al. 2017 (also used for other variables such as sea level), where near-global Argo-period data are re-sampled according to historical data locations, the re-sampled data are mapped and then compared with Argo-period data. In this way, how well the signals in the data-sparse regions can be reconstructed can be evaluated. In Fig. 3 of Cheng et al. 2017, the uncertainty quantification due to mapping is comparable to that of this study.

We acknowledge this issue explicitly in the final section "An additional caveat of this study is that the mapping uncertainty is quantified with the IAP ensemble approach in a

different framework than other sources. We acknowledged the challenge of quantifying sampling/mapping uncertainty: the truth in data gaps will not be known, and a mapping approach is used to fill the gaps.".

Are there any flaws in the data analysis, interpretation and conclusions? Do these prohibit publication or require revision?

While there are no major omissions that call the results into question, some issues warrant further review:

The authors acknowledge the limitation of using only two quality control systems, which suggests that uncertainty from quality control is underestimated or not adequately specified. The two systems are not independent (the CODC was developed by the same group), so potential bias must be discussed. Secondly, using only the IAP's EnOI for mapping uncertainty, even with cross-validation of resampling, raises a concern as the IAP's mapping is also the central estimate. The authors should provide more extensive commentary on whether this leads to an underestimation of uncertainty mapping.The discussion correctly notes that the method cannot capture the 'unknown unknowns', but this is only mentioned briefly. Given that this paper emphasises accuracy (e.g. ~10 ZJ, the best estimate since 2015), this limitation deserves greater emphasis and a lower bound should be provided wherever possible. In Figure 2b, the observed increase is attributed exclusively to quality control. However, a thorough analysis of its origin might provide a clearer picture. For example, if one of the two quality control systems were removed, would the effect on the uncertainty trend decrease? In Equation 1 for the covariance of the error, no reference is made to the statistical methodology. A brief explanation would help to make the method easier to reproduce. Points 1–4 of the discussion are useful and could be expanded upon.

Re: Thanks, and we will answer QC and mapping questions separately:

(1) For WOD and CODC QC, they are very much independent. WOD-QC was developed by the NOAA/NCEI group in the US (Garcia, H. E. et al 2023); CODC-QC was developed by the author group in China (Tan et al. 2023). The key modules in the two systems differ substantially. Still, only two systems are used, different from other uncertainty sources, these limitations have been discussed in the manuscript.

(2) That is a good idea to test if one of the two QC systems is removed. Please find the result in the figure below (Fig. X2). It shows 1) WOD-QC ensemble (members using only WOD-QC) always show larger [5, 95] % spread than CODC-QC-ensemble, indicating CODC-QC is more stable (less sensitive to other approaches); 2) The increase in uncertainty since ~2015 has been reduced substantially, suggesting QC is mainly responsible for this trend. Other reasons include XBT, mapping and vertical resolution.

    Some discussions have been added in the revised manuscript "An additional test using removing one of the two QC systems suggests a substantial reduction of the uncertainty range (e.g., CODC-QC only ensemble spread is ~10 ZJ within 2015-2023, compared with total uncertainty range of ~19 ZJ) and a reduced increasing trend since ~2015, confirming that QC is major responsible for the recent uncertainty increase.".

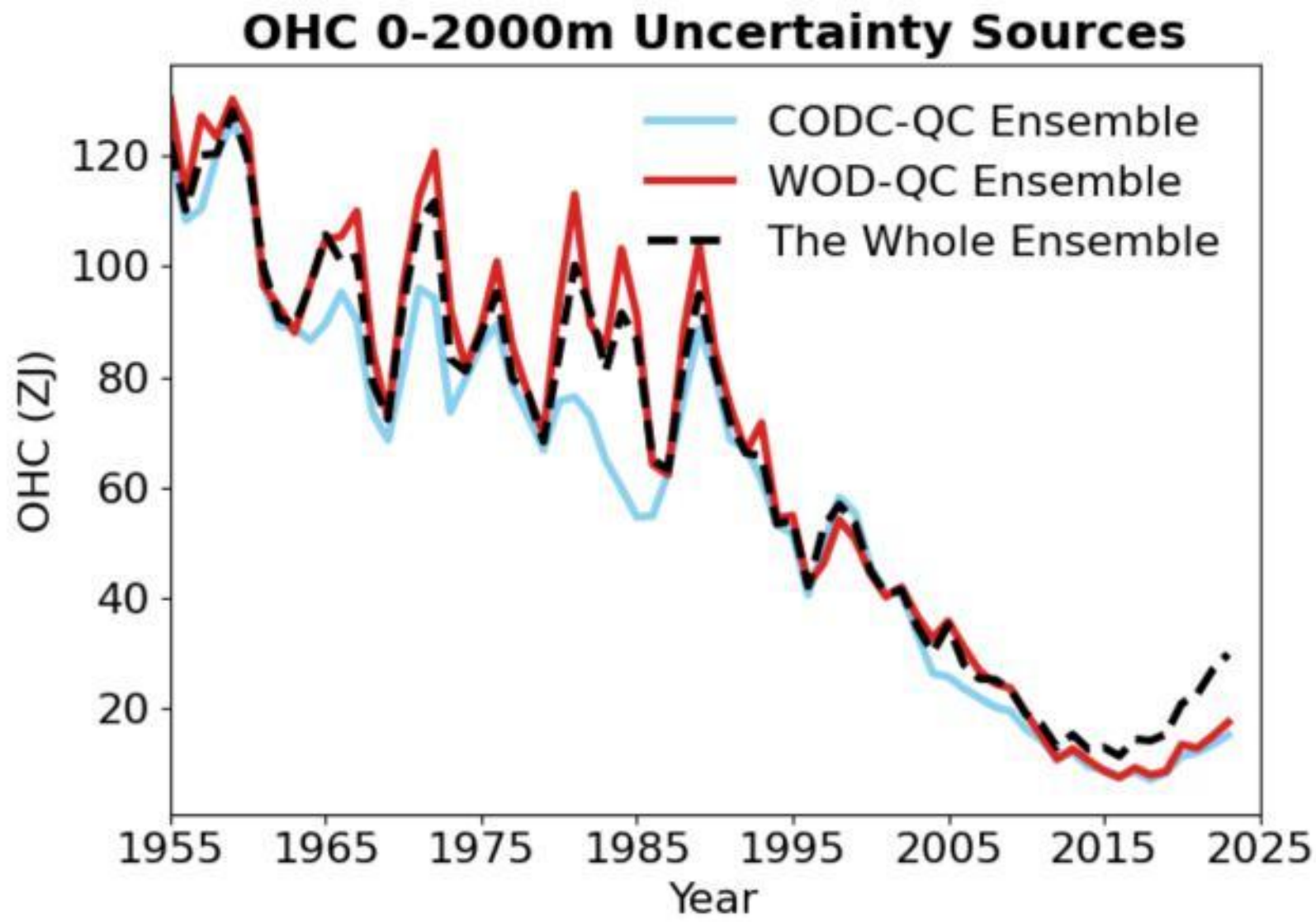

Figure X2. [5, 95] % CI for CODC-QC ensemble (removing all members using WOD-QC), WOD-QC ensemble and all ensemble respectively.

(3) For Eq.1, we have added some information on the covariance, as pointed out by reviewer#1: “Throughout this study, the 'covariance' includes both linear and nonlinear relationships among variables”. And the method for “variance-covariance matrix” is provided in Ablain et al. 2019, which is added in the paragraph after Eq.1. The calculation of Eq.1 is also very straightforward, just multiple calculations of variances, and in the revised manuscript, all codes are available, which we believe ensures the transparency of the calculations in this study.

(4) We have expanded the final (1)~(4) suggestions but not in a conservative way because the overall words limit of the journal.

Is the methodology sound? Does the work meet the expected standards in your field?

The methodology is robust and sophisticated. Its main strengths are as follows: The large ensemble approach utilises three leading techniques for error sources and is well-documented and computationally feasible. This avoids the artificial inflation of error caused by outdated methods. The separation of uncertainty estimates for the annual mean OHC, the trend and the dOHC/dt is methodologically significant and has been correctly implemented. The variance–covariance matrix framework (Equation 2) for time-correlated errors is rigorous and appropriate, and the use of LOWESS for trend estimation with matrix method cross-validation is considered best practice in this field. The decision to limit the number of ensemble members to ~900 (rather than the full 4,374) is well justified by the demonstrated convergence. One comment on the methodology is that selecting three methods per error source implies that three samples are enough to adequately represent the distribution of each source. While this may be justified for some methods, such as vertical regression and climatology, based on the

available literature, for others, such as XBT correction, a more rigorous selection process is required. However, the authors do address this in the 'Methods' chapter.
Re: Thanks. We'd be happy to provide more justifications. Just a note for XBT correction schemes, there are indeed more than 10 available (Cheng et al. 2016). Cheng et al. (2018) conducted an inter-comparison study to evaluate these schemes and found that the three schemes perform best, which are selected in this study. Another reason for selecting the three is: most of the other methods are incomplete in modeling XBT bias: many of them only assume XBT has depth bias but neglect the pure temperature bias. Thus, the three approaches represent the best knowledge we have for XBT biases, and their difference could approximately represent the uncertainty.

Is there enough detail provided in the methods for the work to be reproduced? It is partially satisfactory, but improvements are needed. The raw in-situ data (WOD/CODC) and CERES products are publicly available and cited. The dataset is also available on the IAP FTP server, which is commendable. However, the main shortcoming is the unavailability of the code. The mapping code (IAP EnOI) is not documented sufficiently to allow reproduction. The dataset construction process, which incorporates eight error sources across 908 members, should have an associated code repository. While the article states that the codes for alternative mapping approaches 'are not publicly available', the availability of the authors' own code is not explicitly mentioned. Certain parameters (e.g. the set of 30 CMIP5 models used in EnOI and the climatology construction details for each of the three methods) are mentioned, but not described with enough detail to allow independent reproduction. For full reproducibility, a supplementary methods section with greater technical depth would be necessary.
Re: Thanks, we have put all the codes used to create the analyses in this study from the large-ensemble data available. All codes for figures are available, including both main text figures and Supplementary figures.

The IAP-mapping itself is described extensively in Cheng & Zhu (2016), Cheng et al. (2017), and Cheng et al. (2024) papers. We have provided all these references in this study, which we feel are sufficient, as this paper focuses on the large-ensemble dataset and the uncertainty it yields.

I recommend accepting the manuscript after making the following minor revisions.
1) A statement must be included regarding the availability of the code.
Re: Done.

2) The fact that both systems originate from the IAP/CAS must be explicitly mentioned in relation to the independence of quality control.
Re: Clarified, see previous reply.

3) The restriction to using only one method for matching should be clarified more clearly in the abstract and conclusions.

Re: Probably you mean the mapping method. We added this in both the abstract and the conclusion.

4) The CODC-QC claim regarding 'best available knowledge' should be supported by independent validation.

Re: This sentence has been removed. As in previous reply, we agree that it needs a thorough study and this study only provides some analyses to show their differences (Fig. S5, S6).

5) Standardise abbreviations, expand the statistical basis of equation 1 and qualify the phrase 'best estimate' for the 10 ZJs to reflect remaining structural uncertainties. Re: We agree that "best estimate" for 10ZJ might not be fully justified, given there is no comprehensive evaluation on WOD-QC and CODC-QC. Thus, this sentence has been removed. We keep our estimate of ~19 ZJ for the 2015-2023 OHC uncertainty.

References

1. Cheng, L. & Zhu, J. Uncertainties of the ocean heat content estimation induced by insufficient vertical resolution of historical ocean subsurface observations. *J. Atmos. Ocean. Technol.* **31**, 1383-1396 (2014).
2. Mayer, M., Loeb, N. G., Lyman, J. M., Johnson, G. C. & Winkelbauer, S. The atmosphere's substantial role in interannual variability of Earth's energy imbalance. *Geophys. Res. Lett*. **53**, e2025GL119833 (2026).
3. Lyman, J. M. & Johnson, G. C. Estimating annual global upper-ocean heat content anomalies despite irregular in situ ocean sampling. J. Clim. 21, 5629–5641 (2008).
4. Garcia, H. E. et al. World Ocean Database 2023: User's Manual. NOAA Atlas NESDIS 98 (National Oceanic and Atmospheric Administration, 2024).
5. Tan, Z., Cheng, L., Gouretski, V., Liu, C. & Zhu, J. A new automatic quality control system for ocean profile observations and impact on ocean warming estimate. *Deep Sea Res. Part I: Oceanogr. Res. Pap*. **194**, 103961 (2023).
6. Ablain, M., Meyssignac, B., Zawadzki, L., Jugier, R., Ribes, A., Spada, G., Benveniste, J., Cazenave, A., and Picot, N. Uncertainty in satellite estimates of global mean sea-level changes, trend and acceleration, *Earth Syst. Sci. Data*, **11**, 1189–1202 (2019).
7. Cheng, L. et al. How well can we correct systematic errors in historical XBT data? *J. Atmos. Ocean. Technol*. **35**, 1103–1125 (2018).
8. Cheng, L. & Zhu, J. Benefits of CMIP5 multimodel ensemble in reconstructing historical ocean subsurface temperature variations. *J. Clim*. **29**, 5393–5416 (2016).
9. Cheng, L., Trenberth, K. E., Fasullo, J., Boyer, T., Abraham, J. & Zhu, J. Improved estimates of ocean heat content from 1960 to 2015. *Sci. Adv*. **3**, e1601545 (2017).

0. Cheng, L. et al. IAPv4 ocean temperature and ocean heat content gridded dataset. *Earth Syst. Sci. Data* **16**, 3517–3546 (2024).
